# Decoding the complete organelle genomic architecture of *Stewartia gemmata*: an early-diverging species in Theaceae

**DOI:** 10.1186/s12864-024-10016-8

**Published:** 2024-01-25

**Authors:** Daliang Liu, Zhihan Zhang, Yanlin Hao, Mengge Li, Houlin Yu, Xingruo Zhang, Haoyang Mi, Lin Cheng, Yiyong Zhao

**Affiliations:** 1 Henan International Joint Laboratory of Tea-Oil Tree Biology and High-Value Utilization, College of Life Sciences, Xinyang Normal University, Xinyang, 464000 China; 2 Key Laboratory of Functional Agriculture in Higher Education of Guizhou Province, College of Agriculture, Guizhou University, Guiyang, 550025 China; 3 State Key Laboratory of Public Big Data, College of Computer Science and Technology, Guizhou University, Guiyang, 550025 China; 4 College of Engineering and Technology, Northeast Forestry University, Harbin, 150040 China; 5 Department of Biochemistry and Molecular Biology, University of Massachusetts Amherst, Amherst, MA 01003 USA; 6 Present address: Broad Institute of MIT and Harvard, Cambridge, MA 02142 USA; 7 Department of Public Health Sciences, University of Chicago, Chicago, IL 60637 USA; 8 Department of Biomedical Engineering, Johns Hopkins University, Baltimore, MD 21205 USA

**Keywords:** *Stewartia gemmate*, Mitochondrial genome, Chloroplast genome, Phylogenetics

## Abstract

**Background:**

Theaceae, comprising 300 + species, holds significance in biodiversity, economics, and culture, notably including the globally consumed tea plant. *Stewartia gemmata,* a species of the earliest diverging tribe Stewartieae, is critical to offer insights into Theaceae's origin and evolutionary history.

**Result:**

We sequenced the complete organelle genomes of *Stewartia gemmata* using short/long reads sequencing technologies. The chloroplast genome (158,406 bp) exhibited a quadripartite structure including the large single-copy region (LSC), a small single-copy region (SSC), and a pair of inverted repeat regions (IRs); 114 genes encoded 80 proteins, 30 tRNAs, and four rRNAs. The mitochondrial genome (681,203 bp) exhibited alternative conformations alongside a monocyclic structure: 61 genes encoding 38 proteins, 20 tRNAs, three rRNAs, and RNA editing-impacting genes, including *ATP6*, *RPL16*, *COX2*, *NAD4L*, *NAD5*, *NAD7*, and *RPS1*. Comparative analyses revealed frequent recombination events and apparent rRNA gene gains and losses in the mitochondrial genome of Theaceae. In organelle genomes, the protein-coding genes exhibited a strong A/U bias at codon endings; ENC-GC3 analysis implies selection-driven codon bias. Transposable elements might facilitate interorganelle sequence transfer. Phylogenetic analysis confirmed Stewartieae's early divergence within Theaceae, shedding light on organelle genome characteristics and evolution in Theaceae.

**Conclusions:**

We studied the detailed characterization of organelle genomes, including genome structure, composition, and repeated sequences, along with the identification of lateral gene transfer (LGT) events and complexities. The discovery of a large number of repetitive sequences and simple sequence repeats (SSRs) has led to new insights into molecular phylogenetic markers. Decoding the *Stewartia gemmata* organellar genome provides valuable genomic resources for further studies in tea plant phylogenomics and evolutionary biology.

**Supplementary Information:**

The online version contains supplementary material available at 10.1186/s12864-024-10016-8.

## Introduction

Theaceae, which is included in the order Ericales, is one of the families with higher biodiversity among angiosperms, with nearly 370 accepted species, including many economically, ecologically, and horticulturally important species [1]. Stewartieae (*Stewartia* and *Hartia*) diverged earliest from Theaceae approximately 20.78 million years ago [1]. *Stewartia gemmata* (*S. gemmata*) is a member of the genus *Stewartia*, predominantly found in the southern provinces of China, such as Hunan, Jiangxi, Fujian, Guangdong, and Yunnan, flourishing in mixed forests at altitudes ranging from 900 to 1,500 m. This species typically reaches a height between four and eight meters, characterized by its smooth and grayish-yellow bark. *S. gemmata* is frequently used for ornamental purposes in horticulture due to its high decorative value as vibrant flowers with aesthetic appeal. Utilized in traditional medicine, the bark, roots, and fruits of this plant offer substantial medico-economic value [2]. As an early-diverging species in Theaceae, exploration of the organelle genome of *S. gemmata* will provide an opportunity to unravel the reticulated evolution history caused by hybridization and supplement the genetic resources of Theaceae species. Investigations with the early diverging clade representative will hold significant implications for understanding tea plant family species relationships and the conservation of genetic resources.

Recently, molecular phylogeny supported that Theaceae was mainly divided into three tribes, Gordonieae, Stewartieae, and Theeae [3, 4]. The published genome species in Theaceae were concentrated in *Camellia sinensis* and oil-tea plants [5–10]. Due to the lack of genomic data in other clades, such as the most divergent clade, the phylogeny and other evolutionary studies within Theaceae are still subject to debate. For example, a previous study based on the *matK* and *rbcL* gene markers supported Gordonieae and Stewartieae as sister groups with weak support [3]. A study based on nrDNA ITS sequences indicated that Gordonieae is the sister group to Theeae [4]. The above phylogenetic studies are based on single or partial loci of the plastid genome with limited phylogenetic informative sites that could not yield a high resolution for tea plant family phylogeny. A recent study based on extensive nuclear gene markers successively showed that Stewartieae and Gordonieae are consecutive sister groups of Theeae, with the earliest divergent clade being Stewartieae, followed by Gordonieae and Theeae [1]. Nevertheless, due to the absence of high-quality genome data for the Stewartieae and Gordonieae tribes, some evolutionary questions have been difficult to answer.

In addition to nuclear genomes, plant organelles possess independent genetic material [11]. Similar to the nuclear genome, they have multiple mechanisms for defense against genotoxicity, DNA repair, and maintenance of genome integrity, which play an extremely important role in the ordinary life activities of plants [12]. The mitochondrial genome is much larger and structurally variable, and the chloroplast genome is unique to plants compared to animal organelles [13]. Mitochondria play a crucial role in cellular energy conversion as well as supply processes. They are present in almost all cells, and the genome size is remarkably diverse in green plants, ranging from 20 kb in green algae to millions of bases in some angiosperm species [14, 15]. The mitochondrial genome contains a large number of exogenous sequences from the nuclear and chloroplast genomes [16], as well as a large number of repetitive sequences. In addition, the large number of repetitive sequences may lead to frequent homologous recombination events [17], resulting in a range of genomic structures. These structures mainly include linear, circular, and multibranched subcyclic complex molecular conformations [18, 19]. Chloroplasts play an indispensable role in photosynthesis and the carbon cycle and are responsible for the conversion of light energy into biomass [20, 21]. The structural composition consists mainly of a large single-copy region (LSC), a small single-copy region (SSC), and two inverted repeat regions (IRs) [22], with the size of the genome ranging from approximately 107 kb in *Cathaya argyrophylla* to approximately 218 kb in *Pelargonium* [22].

Advancements in sequencing technologies, along with the widespread use of third-generation sequencing platforms such as PacBio and Nanopore, have significantly improved the precision of genomic assembly [23, 24]. Technological progress has provided a feasible context for resolving conflicts in phylogenomic studies based on extensive gene marks from organellar or nuclear genomes [25]. Owing to the higher copy number of organellar genomes in plant cells and the small genomes, it is easy to obtain more sequences with high sequencing depth at a relatively lower cost [22, 25]. Specifically, the chloroplast genome, with its matrilineal inheritance pattern, conservative genomic features, prevalence of single copies and smaller genomic size, provides significant advantages for phylogenetic research [26]. The evolutionary relationships of the early-diverging clade of angiosperms have been constructed based on 61 plastid genomes, showcases a practical and efficient application of the technology [27]. Subsequently, the phylogenetic relationships of Viridiplantae have been successfully reconstructed by 360 well-documented plastid genomes, providing another outstanding example of utilization [25]. With the progression of organellar phylogenomic research, there has been a consistent expansion and diversification of studies centered primarily on mitochondrial phylogeny [28, 29]. These studies are increasingly being utilized to clarify species phylogeny, as demonstrated in examples such as Ganoderma (reishi mushroom) [30], Orchidaceae (the orchid family) [31], and *Saposhnikovia divaricate* [32].

*S. gemmata*, an ancient species within Theaceae, possesses substantial economic and medicinal value. As of now, there remains a deficiency in the high-quality assembly and annotation of organelle genomes for representative species within the most early-diverging lineage of the tea plant family. In this study, we assembled and annotated high-quality organelle genomes for *S. gemmata*. It facilitated an exhaustive identification of codon bias within the encoding genes, repetitive sequences, and transposable elements (TEs), subsequently elucidating the structural characteristics of the *Stewartia* organelle genomes. In this study, a comparative analysis was conducted on the organelle genomes, along with an examination of the phylogenetic relationships among these species within the Theaceae family. The release of both mitochondrial and chloroplast genomes of *S. gemmata* will not only enrich the existing organelle genomic resources but also facilitate comprehensive investigations into various facets of the Theaceae family. The acquisition of high-quality organelle genomes from representatives of the earliest diverging lineage within the Theaceae family will substantially contribute to the understanding of the biology of the tea plant family. This value dataset will provide novel insights into the genomic characteristics, phylogenetic relationships, and evolutionary history of Theaceae species, thereby laying an important foundation for further scientific investigations and analyses.

## Result

### De novo assembly and annotation of* S. gemmata* organelle genomes

**Fig. 1:**
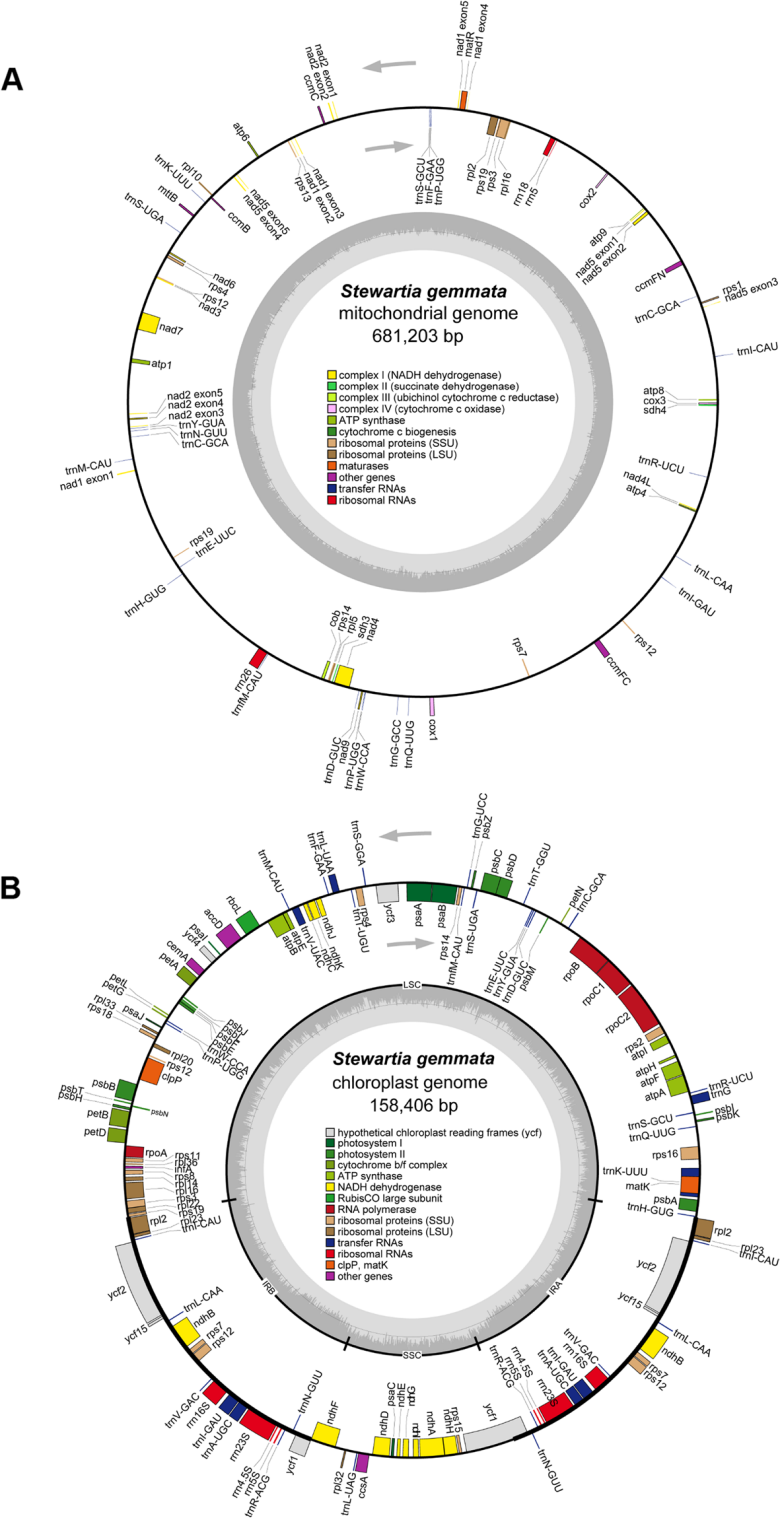
Cartographic representation of the organelle genomes of *S. gemmata*. **A** and **B** represent the mitochondrial genome and the chloroplast genome, respectively. Consistent color blocks are indicative of similar functional gene clusters. The direction of transcription is designated by arrows within the figure: external transcription follows a counterclockwise trajectory, while internal transcription adheres to a clockwise path. In the inner circle bar graph, the dark color indicates GC content, and the light color indicates AT content. In subfigure A, the thick line in the inner circle indicates the two inverted repeats (IRa and IRb, 26,377 bp), which divide the genome into a small single-copy (SSC, 18,136 bp) region and a large single-copy (LSC, 87,516 bp) region

The high-quality organelle genomes of *S. gemmata* were assembled and annotated using a combination of next-generation and third-generation sequencing technologies (Fig. 1). The chloroplast genome manifests a typical monocyclic structure, while the mitochondrial genome exhibits a more complex conformation, encompassing one principal circular structure and two plausible alternative circular conformations (Supplementary Fig. 1). The mitochondrial conformation collectively contains three contigs, specifically contig1 (452.78 kb), contig2 (224.68 kb), and contig3 (1.86 kb), with contig3 as a repetitive sequence (Supplementary Fig. 1A). These contigs collectively signify the complete *S. gemmata* mitochondrial genome sequence. Targeting repetitive regions that may produce multiple assembly conformations, we mapped nanopore platform generated long-read sequences to these regions to validate the possible existence of different mitochondrial genome conformations. We discerned that two conformation pathways were supported by most of the long reads (Supplementary Table 1). The 'contig1-contig3-contig2-contig3' pathway constitutes a singular circular molecule (Supplementary Fig. 1B). The 'contig1-contig3-contig1' and 'contig2-contig3-contig2' pathways represent two potentially existing closed circular alternate molecules, possibly instigated by the repetitive sequence contig3 (Supplementary Fig. 1C). Given that both conformation pathways comprise all genomic information, we defined the singular circular molecule as the primary ring (681,203 bp) for the subsequent analysis (Supplementary Table 2).

The mitochondrial genome of *S. gemmata* was annotated, yielding a total of 61 genes (Supplementary Table 3). The genomic analysis revealed a total of 38 distinct protein-coding genes within the mitochondrial genome, which comprises 24 core genes essential for mitochondrial function and an additional 14 genes that, while not classified as core, contribute to the broader mitochondrial gene repertoire. In the genomic architecture, we have identified a total of 20 tRNA genes, among which two are present in multiple copies, providing insight into the complexity of the organism's translational machinery. Furthermore, the genome encompasses three distinct rRNA genes, essential components of the ribosomal subunits, which play a crucial role in protein synthesis. This configuration of tRNA and rRNA genes underlines the intricacies of the organism's genetic blueprint for RNA-mediated processes. The core genes of the mitochondrial genome of *S. gemmata* comprise five ATP synthase genes, including *atp1*, *atp4*, *atp6*, *atp8*, and *atp9*; nine NADH dehydrogenase genes, namely, *nad1*, *nad2*, *nad3*, *nad4*, *nad4L*, *nad5*, *nad6*, *nad7*, and *nad9*; four cytochrome c biogenesis genes, specifically *ccmB*, *ccmC*, *ccmFC*, and *ccmFN*; and three cytochrome c oxidase genes designated *cox1*, *cox2*, and *cox3*. Additionally, the core genes include a membrane transport protein-encoding gene (*mttB*), a maturation enzyme-encoding gene (*matR*), and a ubiquinol-cytochrome c reductase gene (*cob*). The noncore genes include four ribosomal large subunit genes (*rpl2*, *rpl5*, *rpl10*, *rpl16*), eight ribosomal small subunit genes (*rps1*, *rps3*, *rps4*, *rps7*, *rps12*, *rps13*, *rps14*, *rps19*), and two succinate dehydrogenase genes (*sdh3*, *sdh4*) (Fig. 1A).

The chloroplast genome of *S. gemmata* is a single circular molecule of 158,406 bp with a GC content of 37.26%. The annotation of the chloroplast genome led to the identification of a total of 114 genes (Fig. 1B and Supplementary Table 4), including 80 unique protein-coding genes (eight of which are multiple copies), 30 transfer RNA (tRNA) genes (seven of which exist in multiple copies), and four ribosomal RNA (rRNA) genes (all of which exist in multiple copies). The protein-coding genes belonged to 16 distinct gene families, including 11 NADH dehydrogenase subunit genes, five photosystem I subunit genes, 16 photosystem II subunit genes, six cytochrome b/f complex subunit genes, six ATP synthase subunit genes, and a single large subunit gene for ribulose-1,5-bisphosphate carboxylase/oxygenase. Additionally, there are four DNA-dependent RNA polymerase genes, nine large ribosomal subunit genes, 12 small ribosomal subunit genes, a single maturation enzyme-encoding gene, a cytochrome c biogenesis enzyme-encoding gene, a membrane protein-encoding gene, and a protease gene. There is also one gene for the subunit of acetyl-*CoA* carboxylase, another one for a translation initiation factor, and four for conserved open reading frames.

To assess the quality of organelle genomes, we mapped long-reads and short-reads to the assembled genome and counted the coverage depth of genomic nucleic acid loci. In our study, the mitochondrial genome and chloroplast genome reached a sequencing coverage depths of 300X and 3,200X for short-reads and long-reads, respectively, with 100% genome-wide coverage (Supplementary Fig. 2).

### Variation in organelle genome composition and mutation rate analysis of Theaceae species

We compared the organellar genome of *S. gemmata* with six other Theaceae species and two species (*Aegiceras corniculatum* and *Diospyros oleifera*) from closely related families within the order Ericales. In the mitochondrial genome, the gene composition of *S. gemmata* differs significantly from that of other species. We observed two main differences in the mitochondrial genome of *S. gemmata* compared to other species: first, only a small number of genes possess duplicated copies (Supplementary Fig. 3); second, there is evidence of gene loss and gain, particularly in tRNA genes. The substantial variability observed in the genes among these species implies a complex history of gene loss and gain events throughout Theaceae mitochondrial genome evolution. The gene compositions across the species examined in the chloroplast genome were relatively conserved (Supplementary Fig. 4). However, there also seems to be a pattern of tRNA gene gain and loss in certain species, further demonstrating the dynamic nature of organellar genome evolution.

**Fig. 2:**
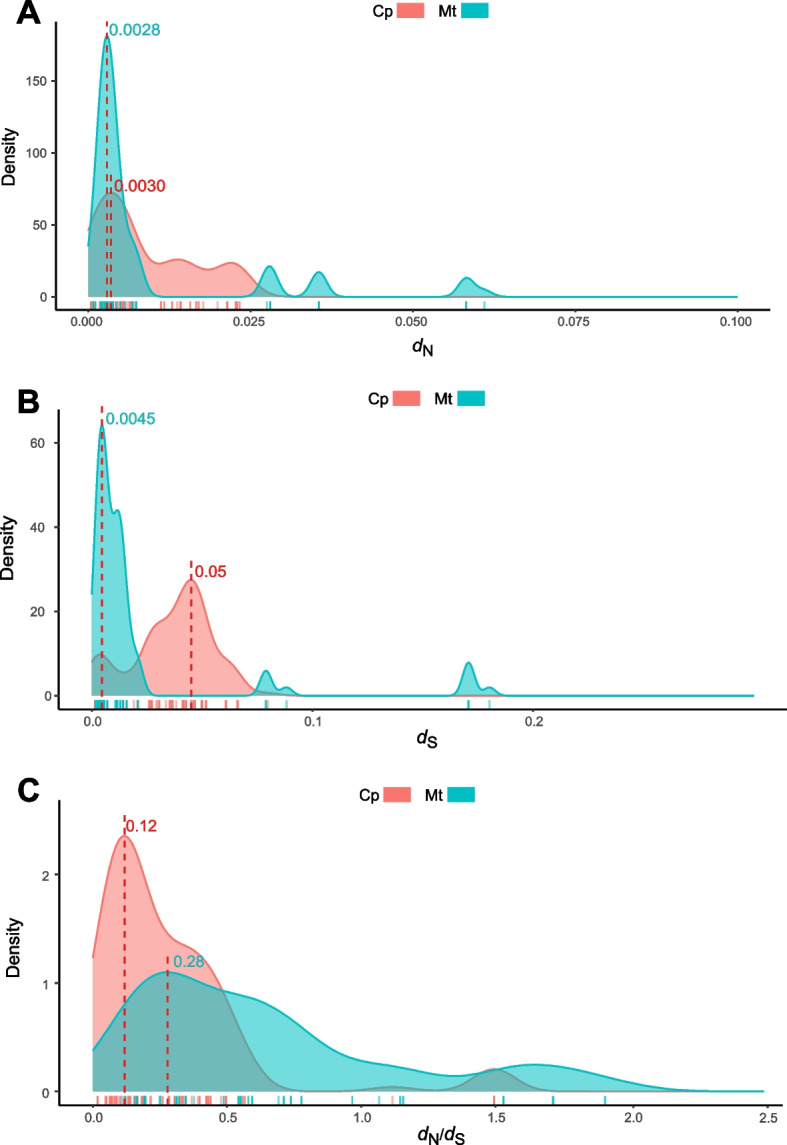
Graphical representation of *d*_N_, *d*_S_, and *d*_N_/*d*_S_ values of paired orthologous genes in the organelle genomes of seven species of Theaceae. **A**, **B**, and **C** show the density distribution of the values of *d*_N_ and *d*_S_ and the ratio of *d*_N_/*d*_S_, respectively. The numbers in the graph indicate the peak value. Red indicates the chloroplast genome (Cp), and blue indicates the mitochondrial genome (Mt)

In addition, to further resolve the evolutionary rate differences in the organelle genomes of Theaceae, we analyzed the evolutionary rates of protein-coding genes shared by the species of Theaceae in the phylogenetic tree. We found that the *d*_N_ and *d*_S_ values of genes in chloroplasts were greater than those of mitochondrial genes (Fig. 2A-B), which may be due to different evolutionary patterns. From the trend of *d*_N_/*d*_S_, the value of chloroplasts is approximately 1/2 of that of mitochondria, suggesting that the chloroplast genome may have been subjected to a higher degree of negative selection and is more conserved at the protein sequence level (Fig. 2C).

### Characteristics of codon usage bias of protein-coding genes in the *S. gemmata* organellar genome

**Fig. 3:**
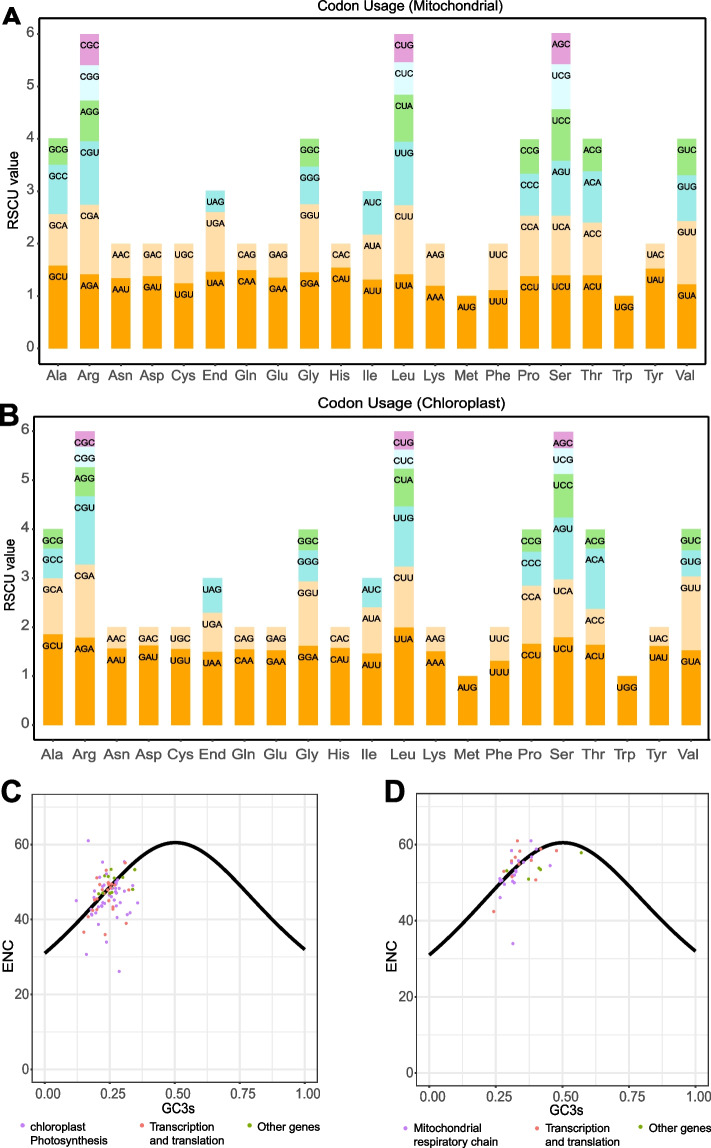
Summary of codon characteristics of protein-coding genes in the *S. gemmata* organelle genome. **A** and **B** represent the relative synonymous codon usage (RSCU) preference distribution maps for the mitochondrial and chloroplast genomes. The horizontal coordinates represent the 21 amino acids, and the vertical coordinates are the values of RSUC. **C** and **D** indicate a projection of ENC against GC3s based on protein-coding genes in chloroplasts and mitochondria, respectively. The solid line within the figure signifies the expected trendline of gene positions given codon usage solely driven by GC3s composition. Purple dots indicate genes related to photosynthesis (**C**) and respiratory chain (**D**), red dots indicate transcription and translation-related genes, and green dots indicate other genes

To investigate the codon usage bias characteristics of the *S. gemmata* organelle genomes, we analyzed the relative synonymous codon usage (RSCU) values of all PCGs (Fig. 3A-B, Supplementary Table 5). A certain degree of variation was observed in the frequency of codon usage between the different organelles. The range of RSCU values in the chloroplast spanned from 0.32 (CGC in arginine) to 1.99 (UUA in leucine), whereas in the mitochondria, the range was from 0.46 (CAC in histidine) to 1.58 (GCU in alanine). Only AUG (start codon) and UGG had RSCU values of 1 in both organelles, and the rest were either greater than or less than 1, suggesting a general codon preference for PCGs. There are 31 codons with RSCU values greater than 1 in the mitochondrial and chloroplast genomes, of which only three end in G/C and the rest end in A/U, suggesting that there is a strong A/U tendency in the use of codons in the genomes of *S. gemmata* organelles.

Scatter plots of ENC-GC3 are frequently employed to estimate factors influencing codon usage patterns [33]. We unveiled that the two organellar genomes exhibit similar bias patterns, with a minority of genes positioned on or close to the standard curve, while the majority demonstrate deviations from this curve (Fig. 3C-D). Remarkably, the genes exhibiting pronounced deviations in chloroplasts predominantly pertain to photosynthesis (Fig. 3C), while those in mitochondria are primarily associated with respiration (Fig. 3D). Existing research suggests that codon bias in genes with effective codon counts (ENC) less than 35 are more susceptible to mutation, whereas genes with effective codon counts greater than 35 are more likely to be the result of artificial or natural selection [34]. We found that the mitochondrial genes *atp9*, *trnI*, and *trnW* have ENC values of 33.99, 28.5, and 22.45, respectively, all less than 35. Among them, *atp9* also had values less than 35 in other reports [30]. In the chloroplast genome, genes with ENC values under 35 included *psbj* (33.97), *psbl* (26.15), *petN* (30.7), *trnR-ACG* (32.95), *trnF-GAA* (23.83), and *trnW-CCA* (22.45) (Supplementary Table 6).

### Identification of putative RNA editing sites of PCGs in the mitochondrial genome

**Fig. 4:**
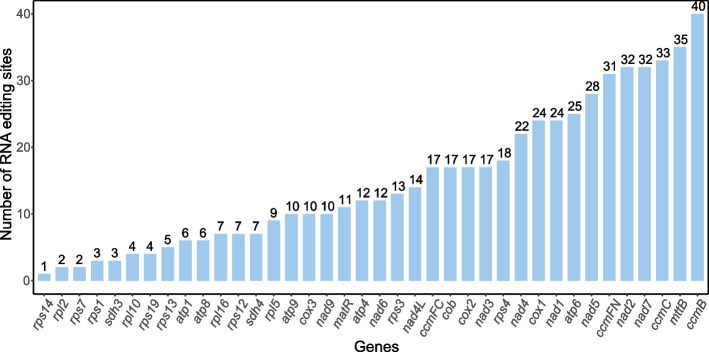
Identified traits of RNA editing sites in protein-coding genes of *S. gemmata* mitochondrial genomes. The X coordinate indicates PCG, and the Y coordinate indicates the number of RNA editing sites

RNA editing plays an essential role in the modulation of genes in plant mitochondrial genomes [35]. To obtain more precise results, we retained potential RNA editing sites with probability values greater than 0.9. We discerned 570 potential RNA editing sites in 38 mitochondrial PCGs of *S. gemmata* (Fig. 4), more than previously identified in *Arabidopsis thaliana* (441) and *Brassica* (427) mitochondrial genomes [36]. The putative editing sites all involved a base change from C to U, consistent with earlier studies [35, 37]. Among mitochondrial genes, the *ccmB* gene was identified with 40 potential RNA editing sites and contained the most potential RNA editing sites in our analyses. The *mttB* gene follows with 35 RNA editing occurrences. Most RNA editing instances lead to alterations in the amino acid sequence, notably causing the production of initiation and termination codons, thereby modifying the gene structure. In our data, we identified seven such genes: *atp6* and *rpl16* acquired termination codons, while *cox2*, *nad4L*, *nad5*, *nad7*, and *rps1* obtained initiation codons. In the case of *cox2*, it is affected by editing events at two positions (Supplementary Table 7).

### Repeated sequence analysis of *S. gemmata* organelle genomes

**Fig. 5:**
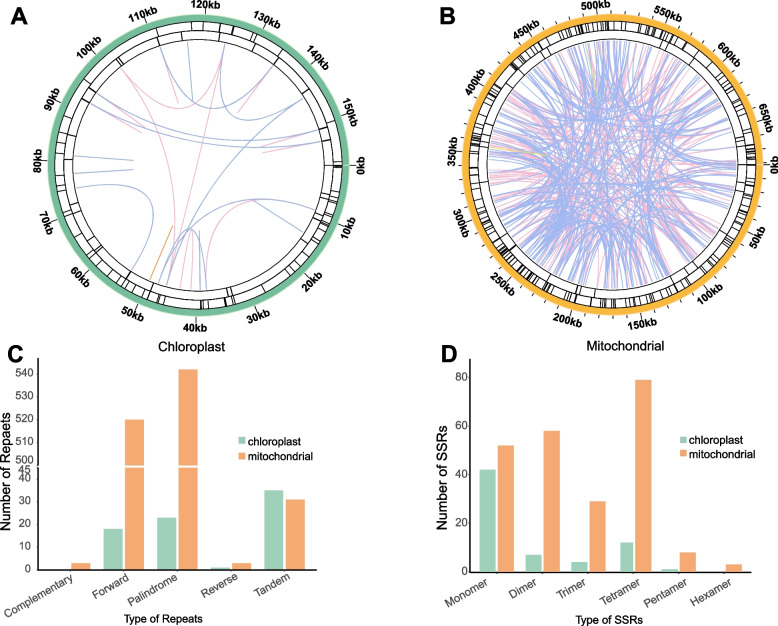
Repeat sequences within the mitochondrial and chloroplast genomes. The internal circle-colored lines correlate two dispersed repeats: blue for palindromic repeats, pink for forward repeats, yellow for reverse repeats, and green for complementary repeats. The second circle's black line denotes tandem repeats, while the outermost circle designates microsatellite repeats. **A** and **B** represent the physical distribution of repetitive sequences in the mitochondrial genome and the chloroplast genome, respectively. **C** and **D** represent the respective types of repeats and simple sequence repeats (SSRs)

Plant organellar genomes are replete with repetitive sequences, primarily categorized into two types, tandem and dispersed repeats, which are differentiated principally by their physical proximity [38]. We conducted a genome-wide annotation of repetitive sequences to decipher the characteristics of *S. gemmata* repetitive sequences and their potential influence on genome structure (Fig. 5). Microsatellites or simple sequence repeats (SSRs), a particular class of tandem repetitive sequences, typically range in length from 1–6 bp. We identified 229 and 66 SSRs in the mitochondrial and chloroplast genomes, respectively (Fig. 5). Among these SSRs, 42 mononucleotide SSRs (accounting for 63% of the total) were detected in the chloroplast genome, all of which were A/T type, indicating a strong bias. The numbers of dimers, trimers, tetramers, and pentamers in chloroplast were 7, 4, 12 and 1, respectively. However, hexamer SSRs were not detected. In contrast, the distribution of SSRs in the mitochondrial genome was more balanced, with tetramers being the most abundant (74, approximately 34%), followed by monomers and dimers, accounting for 22% and 25%, respectively. Mononucleotide repeat sequences were exclusively A/T bases (Fig. 5D, Supplementary Table 8).

In the *S. gemmata* mitochondrial genome, 31 tandem repeats have been detected, ranging from 11 to 39 bp in length. In the chloroplast genome, a total of 35 tandem repeats were identified, displaying a variation in length ranging from 9 to 33 base pairs (bp), as illustrated in Fig. 5C and detailed in Supplementary Table 9. In addition to these tandem repeats, dispersed repeats are also prevalent throughout the genome, and their function is observed to be of equivalent significance, as supported by a previous study [39]. We detected 1026 and 42 pairs of dispersed repeats in the mitochondrial and chloroplast genomes, respectively (Fig. 5A-C and Supplementary Table 10). Among the four types of dispersed repeats, forward, reverse, complementary, and palindromic, complementary repeats were not found in the chloroplast genome, and the other types of repeats were distributed in both organellar genomes, with most being approximately 30 bp in length.

### Detection of lateral gene transfer (LGT) between organelles and TE annotation

**Fig. 6:**
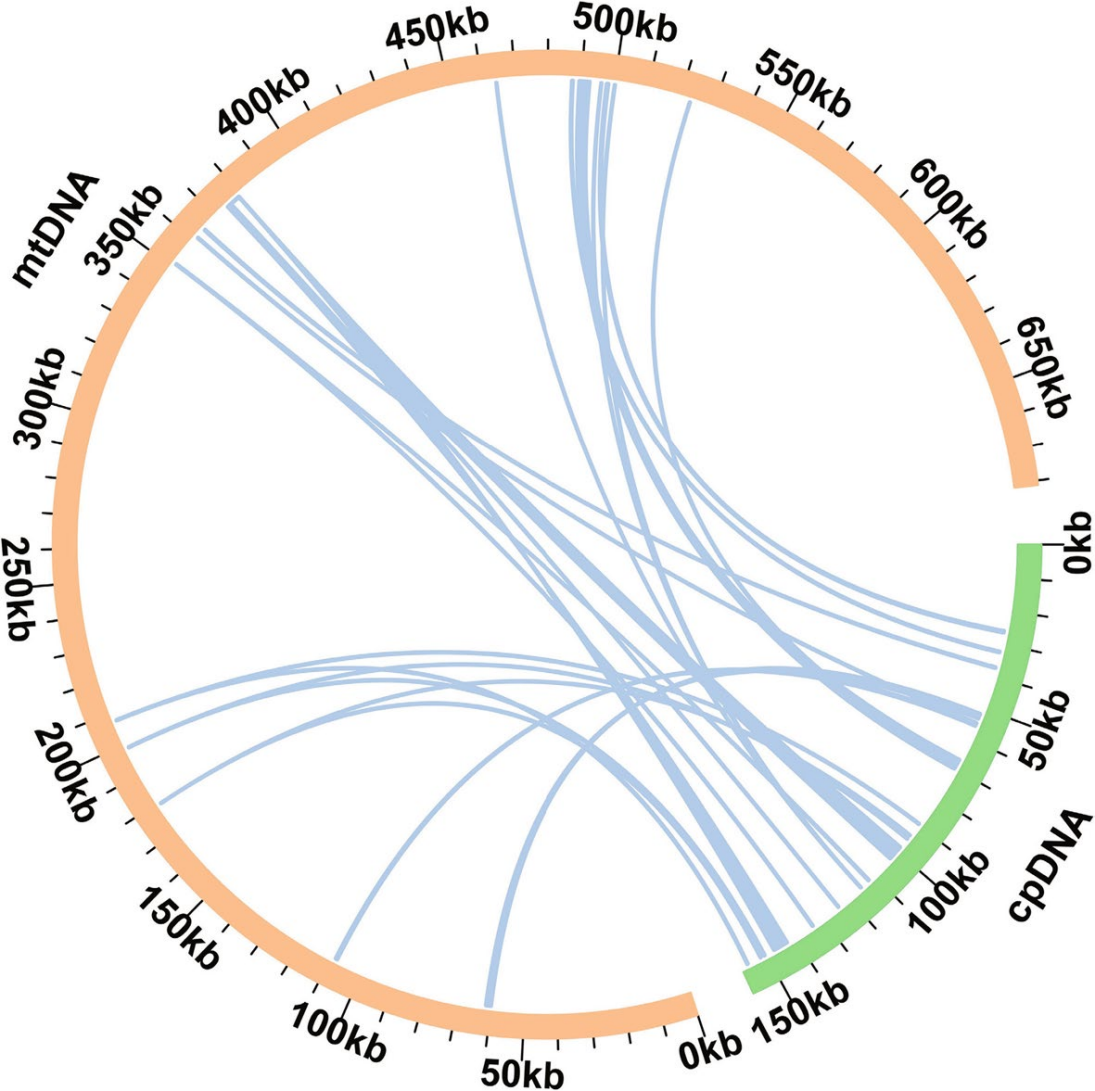
Comparative homology analysis of organelle genome sequence data. The mitochondrial and chloroplast genomes are represented by yellow and green arcs, respectively, while the blue lines connecting the arcs represent homologous genomic fragments

Homology analysis of the *S. gemmata* mitochondrial and chloroplast genomic sequences revealed 17 fragments with an identity greater than 0.8, totaling a length of 9,025 bp, constituting 1.32% of the total mitochondrial genome length (Fig. 6 and Supplementary Table 11). Three homologous fragments were over 1,000 bp, specifically MTPT1, MTPT15, and MTPT17, with MTPT1 being the longest (4,396 bp), corresponding to two positions of IRa and IRb in the plastid genome. These 17 homologous sequences contained 32 gene sequences from the plastid genome. Of these, 15 were complete, including nine protein-coding genes (*psbJ*, *psbL*, *psbF*, *psbE*, *petL*, *petG*, *ndhJ*, *ndhK*, *rps7*) and six tRNA genes (*trnD-GUC*, *trnN-GUU*, *trnI-CAU*, *trnM-CAU*, *trnP-UGG*, *trnW-CCA*).

To validate the sequences of the MTPT regions in the mitochondrial genome, we selected nine MTPT regions with a length greater than 80 bp and mapped the mixed long reads from the mitochondrial and chloroplast genomes to corresponding areas. The mitochondrial reads were mapped to the MTPT and its flanking regions, whereas the chloroplast reads could only be mapped to the MTPT region, demonstrating that the sequence of the corresponding MTPT region had been integrated into the mitochondrial genome (Supplementary Fig. 5).

The numbers of TE elements in the mitochondrial and chloroplast genomes were 133 and 58, respectively, with total lengths of 1,9427 and 8,741 bp (Supplementary Tables 12 and 13). These were mainly of two types: DNA transposons (*EnSpm*/*CACTA*, *Harbinger*, *Helitron*, *Mariner*/*Tc1* (specific to the mitochondrial genome), *MuDR*, *hAT*) and retrotransposons (*Copia*, *Gypsy*, *L1*). Mitochondrial genome TEs were predominantly retrotransposons (88 fragments, 16,158 bp), while those in the chloroplast genome were mainly DNA transposons (41 fragments, 6,292 bp). Finally, we observed a partial overlap between the sequence coordinates of the MTPT region and some TE elements in the organelle genomes (Supplementary Table 14).

### The utilization of the organelle genomes of *S. gemmata* facilitated phylogenomic analyses of the tea plant family

**Fig. 7:**
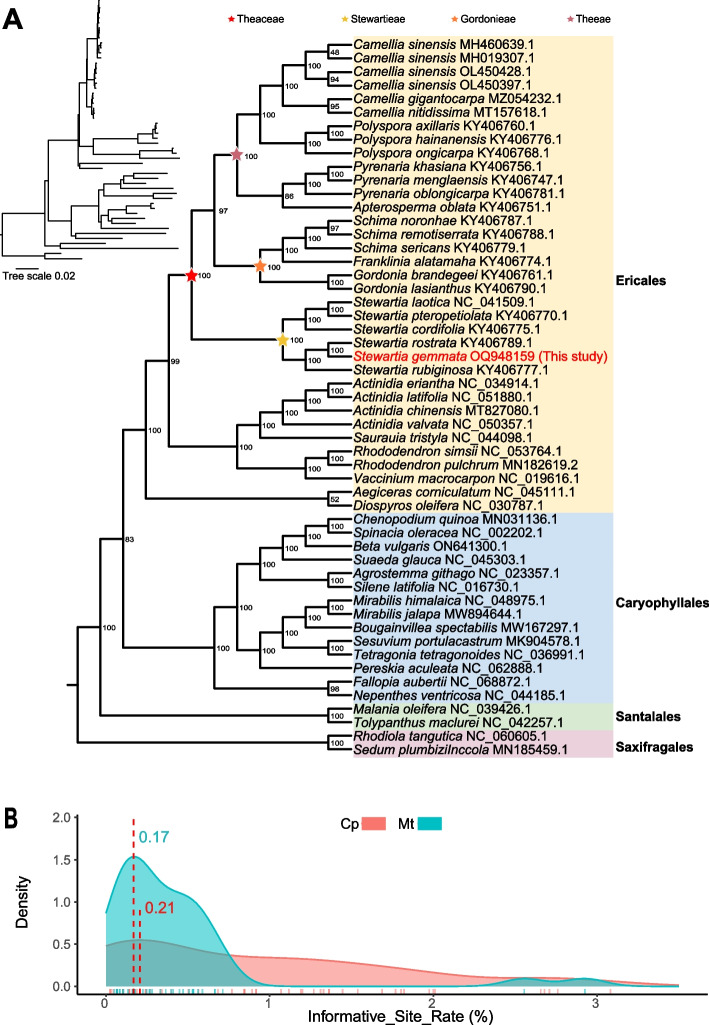
Best maximum likelihood phylogenetic tree based on chloroplast genomes of 53 species (including 25 species of Theaceae). **A** shows the phylogenetic tree constructed by the chloroplast genome. Numbers above each branch are the maximum likelihood bootstrap of each clade > 50%. The four different background colors indicate different orders, respectively. The red font indicates the species of this study. In the top left corner is a tree with branch length information. Pentagrams of different colors at the nodes represent the Theaceae family and delineate its three distinct tribes. **B** shows the information loci of organelle genes involved in the construction of phylogenetic relationships (number of informative loci/effective length of the gene). Red indicates the chloroplast genome (Cp), and blue indicates the mitochondrial genome (Mt)

To gain a more comprehensive understanding of the phylogenetic relationships of the Theaceae species, we constructed a maximum likelihood (ML) phylogenetic tree based on 36 mitochondrial genomes and 53 chloroplast genomes, including the *S. gemmat*a released in this study*,* with two species of Saxifragales as outgroups (Supplementary Table 15). The ML phylogenetic trees based on mitochondrial and chloroplast genome sequences strongly supported Santalales and Caryophyllales as successive sister groups to Ericales (Fig. 7A and Supplementary Fig. 6), the species of Theaceae are monophyletic, with *S. gemmata* at the early-diverging lineage of the Theaceae family. In the phylogenetic reconstruction based on chloroplast genomes, the Theaceae family distinctly segregates into three tribes. Within this framework, the Stewartieae tribe emerges as a sister group to a clade formed by the Gordonieae and Theeae tribes. Our phylogenetic analysis supports the monophyly of several genera within this family, specifically *Camellia*, *Polyspora*, *Pyrenaria*, *Schima*, *Gordonia and Stewartia*. In the Gordonieae tribe, *Gordonia* and *Franklinia* occupy successive sister positions relative to *Schima*. Within the Theeae tribe, *Apterosperma* is delineated as the sister group to *Pyrenaria*, while *Camellia* and *Polyspora* also exhibit a sister group relationship. However, The limited availability of mitochondrial genome sequences from Theaceae constrains our understanding of their evolutionary dynamics from mitochondrial gene markers. This gap impedes a thorough exploration of the mitochondrial genome's influence on the family's phylogeny (Supplementary Fig. 6). In addition, we also found subtle conflicted relationships between the mitochondrial and chloroplast phylogeny in the genus *Camellia*. Through a comprehensive analysis of the informative sites within the orthologous genes specific to the genus *Camellia*, we discerned a discrepancy in the informative site rates between the chloroplast and mitochondrial genomes. Averagely, the informative site rate associated with the chloroplast genome was observed to be higher than that of the mitochondrial genome (Fig. 7B). Based on these findings, we hypothesize that the phylogenetic tree constructed from the chloroplast genome may provide a more accurate and representative depiction of the underlying evolutionary relationships for the tea plant family.

### Analysis of the mitochondrial genome collinearity among *S. gemmata* and other species of Theaceae

**Fig. 8:**
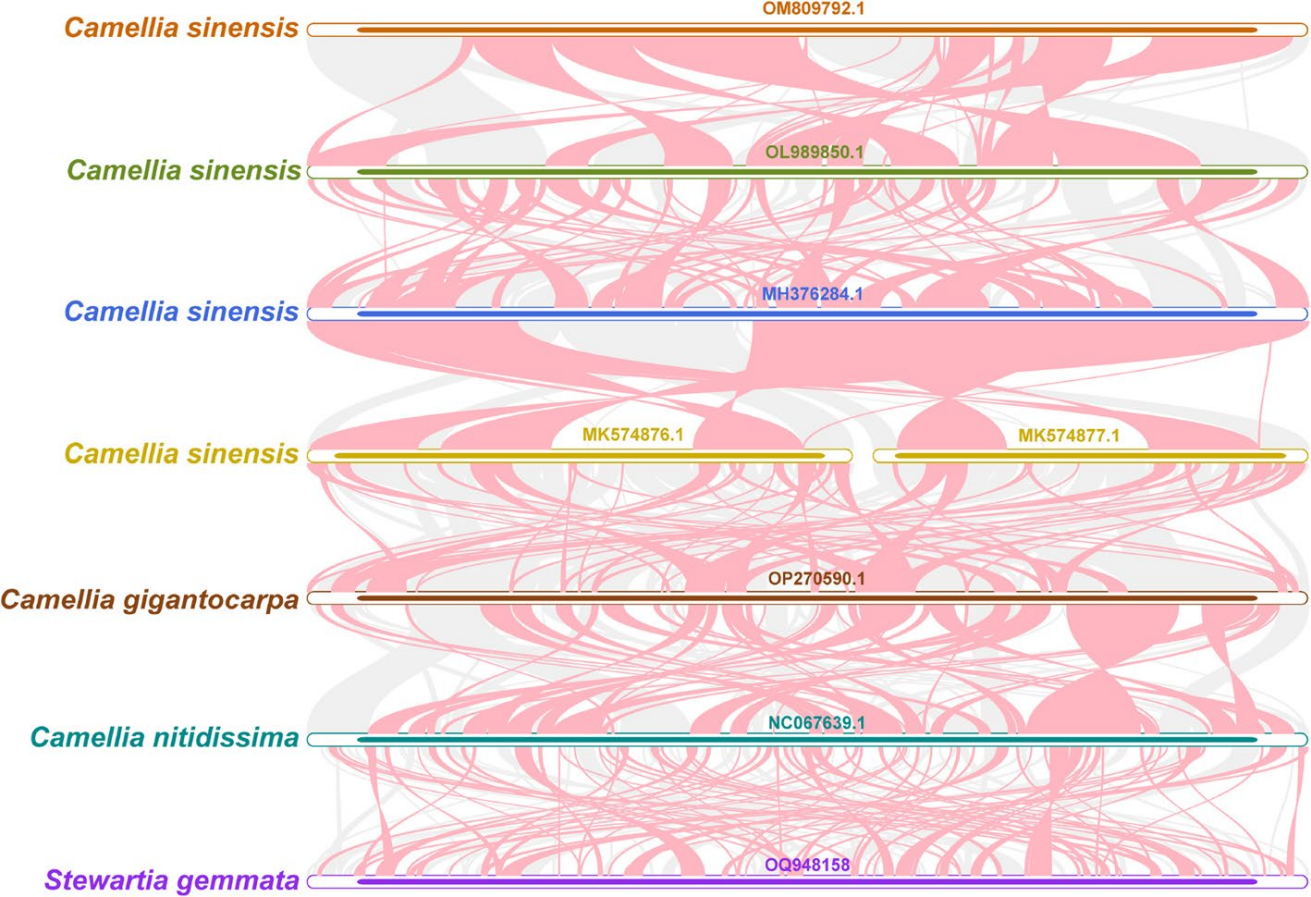
Mitochondrial genome collinearity analysis of seven selected *Camellia* species. Red arc-shaped regions mark areas of inversion occurrence, and gray sections represent those with a high degree of homology

To better elucidate the conservatism of mitochondrial genome evolution between *S. gemmata* and other species in Theaceae, we performed a collinearity analysis of the mitochondrial genome sequences. The results showed that a total of 5,968 regions of covariance existed among the seven Theaceae species (Fig. 8 and Supplementary Table 16), with a wide range of collinearity. Among them, the total length of the covariance blocks between OP270590.1 and OL989850.1, OL989850.1 and OM809792.1, MH376284.1 and MK574876-7.1, and MK574876-7.1 and NC067639.1 was all more than 1000 kb. At the same time, we were able to identify a large number of inverted regions in the genomes of these species, suggesting that the chromosomal structure of the mitochondrial genome is less conserved.

## Discussion

### Structure, size, and repetitive sequences in the organelle genomes of *S. gemmata*

A prior investigation revealed that while chloroplast genomes exhibit conserved size and gene structure across cucurbit species, mitochondrial genomes display considerable heterogeneity. This diversity encompasses variations in size, gene content, organizational structure, and noncoding DNA characteristics [40]. This is the first study to report the complete organelle genome of *S. gemmata*. Considering the long-read sequencing coverage of repeated nodes, we inferred two mitochondrial genome conformations, including a closed circular and two small circular alternative conformations mediated by the repeat. Our results indicated that the high percentage of long-read support for alternative conformations implies that the mitochondrial genome of *S. gemmata* possesses a high frequency of recombination. On the other hand, we uncovered low conservation in the genetic composition, especially in tRNA-related genes. The fluctuating trend of total copy numbers, initially increasing and then decreasing, indicates historical genetic expansion events followed by a current contraction phase in the Theaceae mitochondrial genomes.

Typically, the structure of land plant chloroplast genomes exhibits a quadripartite circular conformation composed of a pair of IR sequences and LSC and SSC regions with conservative structural features [22]. We found the chloroplast genomic structures along with gene type and number to be fairly comparable in Theaceae [41]. In line with our observations, we propose that this highly preserved characteristic could potentially be an outcome of evolutionary mechanisms subjected to purifying selection. Such selection dynamics appear to favor the functional conservation of chloroplasts, specifically those involved in photosynthetic processes. This suggests a significant evolutionary advantage in maintaining the efficiency and integrity of photosynthesis-related functions within the chloroplast.

Repetitive sequences are widespread in organelle genomes, including tandem repeats, dispersed repeats, and SSRs, playing a vital role in genomic structure and genome size and could mediate genome recombination [17, 23, 39, 42, 43]. We detected an abundance of multiple types of repetitive sequences in the *S. gemmata* organelle genomes as well. The number of dispersed repeats in the chloroplast genome is significantly lower than that in the mitochondrial genome, which results in a more complex structure of the mitochondrial genome compared to the chloroplast genome. On the other hand, SSRs are often used to develop molecular markers due to their exceptional polymorphism across species. The application of SSRs is widely utilized in species identification, thus facilitating the conservation and utilization of germplasm resources [44]. In this study, we identified a substantial number of SSRs, providing a framework for germplasm identification, conservation, and utilization.

### Codon usage pattern analysis among mitochondrial and chloroplast genes of *S. gemmata*

As an important evolutionary phenomenon, codon usage bias in genes has been documented in a variety of organisms in prokaryotic and eukaryotic species [45]. Such biases are attributed to an intricate interplay of mutation pressure, natural selection, and genetic drift over long periods of evolutionary time [46, 47]. Extensive studies have been conducted on codon bias in plants, revealing that codon usage is significantly influenced by the composition of nucleic acids in the nuclear genome. In organelle genomes, natural selection has a more pronounced effect on codon bias [33, 48]. We found a strong predilection for A/U-ending codons in the organelle genomes of *S. gemmata,* which is consistent with codon usage bias within the mitochondrial genomes of the other four species in Theaceae [30]. Similar findings were also reported in previous studies on the chloroplast genomes of several plants, including monocots such as wheat and rice [49, 50] and eudicots such as cucumber and *Elaeagnus* [33, 51], which suggests that A/U-ending codon bias might be an inherent characteristic of organelle genomes.

The ENC-GC3 (effective number of codons versus the third position GC content) analysis is a robust technique employed to investigate the underlying factors that may influence species-specific codon usage patterns [33]. This analysis posits that genes located proximally or overlapping with the standard curve within the ENC-GC3 plot are more susceptible to the influence of mutations, potentially altering their codon bias. This relationship allows for an exploration of the evolutionary constraints and mutational pressures acting upon these genes. On the other hand, genes further from the standard curve are more likely influenced by natural selection [34]. Our research revealed that most photosynthesis-related genes in the chloroplast genome, as well as the majority of respiration-related genes in the mitochondrial genome, deviate from the standard curve. This deviation is consistent with the trends seen in other species within the tea plant [30]. This indicates that these genes in Theaceae species are under intensified natural selection pressure, tending toward a more conservative evolution.

### TEs and lateral gene transfer (LGT) in mitochondrial and chloroplast genomes

Transposable elements (TEs) in mitochondria are common in intergenic regions. For instance, TEs are abundantly distributed in the mitochondrial genome of *Arabidopsis thaliana*, suggesting a transfer from nuclear genomes to mitochondrial genomes [52]. In addition, there is evidence of DNA sequence transfer from the plastid genome to the mitochondrial genome without functions [23]. In our study, the TE in the mitochondrial genome was dominated by retrotransposons [53], while the chloroplast genome was dominated by DNA transposons. In addition, we observed an overlap between TE and some MTPTs, especially the DNA-11N_SBi element, shared in the mitochondrial and chloroplast genomes. This suggests that TEs might also be involved in sequence migration between organelle genomes. LGT could play a vital role in elucidating the evolutionary relationship between mitochondrial and chloroplast genome structures. We found 17 mitochondrial plastid DNAs (MTPTs) in the mitochondrial genome, comprising 15 complete chloroplast genes. Prior research identified four plastid-derived tRNA genes (*trnD-GUC*, *trnH-GUG*, *trnN-GUU*, and *trnW-CCA*) in the mitochondrial genomes of Asterids and *Ligustrum quihoui* [53]. Our study detected that *trnD-GUC*, *trnN-GUU*, and *trnW-CCA* originated from the chloroplast genome, which implied that the mitochondrial genome acquired these genes during an ancient LGT event. The longest MTPT1 (4,396 bp) had 100% similarity, indicating a likely recent acquisition from the chloroplast.

### Phylogenetic relationships in Theaceae

A previous study has shown that the tribes Stewartieae and Gordonieae are successive sister groups to the tribe of Theeae [1]. The tribe Stewartieae, as the earliest diverging tribe within the Theaceae family, occupies a pivotal position in phylogenetic research for understanding the evolutionary history of the tea plant family. Unfortunately, there is a lack of a comprehensive and high-quality assembly and annotation of both organelle and nuclear genomes for the Stewartieae and Gordonieae tribes. This absence has created gaps, affecting not only phylogenetic analyses but also various other aspects of genetic and evolutionary studies within the tea plant family. In our study, we released the organelle genomes of *S. gemmata* and reconstructed the phylogeny based on the conserved PCGs of the mitochondrial and chloroplast genomes. The highly supported phylogeny clearly showed that all species were divided into three clades, Santalales, Caryophyllales, and Ericales. The phylogenetic trees from both the mitochondrial and chloroplast genomes suggested that *Stewartia* was the first-diverged clade of Theaceae. The chloroplast-derived phylogenetic relationships within Theaceae are largely congruent with those established in previous studies [54]. The current mitochondrial genome dataset encompasses only species from the *Camellia* genus and the species *S. gemmata* released in this study from the *Stewartia* genus. A limited number of genomes impede our understanding of intergeneric relationships within Theaceae. The acquisition and analysis of additional mitochondrial and nuclear genomes are anticipated to elucidate the phylogenetic relationships of Theaceae species more comprehensively in the future. Moreover, in the phylogenetic trees derived from mitochondrial genomes (ML), regardless of the selection of different gene sets, the tea plants (*Camellia sinensis*) are not a monophyletic group. Utilizing the analysis of phylogenetically informative sites of mitochondrial and chloroplast orthologous genes, we identified that the uncertainty in the relationships within Theeae, as depicted in the mitochondrial maximum likelihood (ML) trees, is primarily attributable to the scarcity of informative loci within mitochondrial genes (Fig. 7B and Supplementary Fig. 6). Moreover, an analysis of organelle sequence transfers revealed that the mitochondrial genome contains exogenous sequences from the chloroplast genome in *S. gemmata*. Differences in the evolutionary mutation rates of sequences from different genomes and mitochondrial-riched recombination might affect the resolution of phylogeny. To date, fewer than ten Theaceae mitochondrial genomes have been sequenced, and the limited number of genomes might lead to data bias, thus limiting the phylogentic resolution to some extent. Overall, our study provides the first mitochondrial genome of the genus *Stewartia* that contributes to the understanding of phylogenetic relationships within Theaceae.

## Conclusion

In this study, we firstly present a comprehensive assembly and annotation of the complete mitochondrial and chloroplast genomes of *S. gemmata*, utilizing both Illumina short-read and Nanopore long-read sequencing technologies. This organelle genome dataset, as a representative of the earliest diverging clade of the tea plant family, lays the foundation for further exploration of multiple biological facets of Theaceae, including phylogeny, evolutionary history, lateral gene transfer (LGT), and complexities of species hybridization. The study characterized different aspects of the organelle genomes, including the genome size, composition, repeated sequences, and LGT events between the organelle genomes. A large number of repetitive sequences have been identified, elucidating the characteristics of the two types of organelle genomes. Additionally, the identification of simple sequence repeats (SSRs) provided data to support the development of molecular phylogenetic markers. The reconstruction of the phylogenetic relationships within the Theaceae family, based on conserved organelle genes, has afforded valuable insights into its phylogeny. This study has elucidated not only the specific characteristics of the organelle genomes of *S. gemmata* but also the broader evolutionary history of the tea plant family. The complete decoding of the organelle genome serves as a pivotal resource, paving the way for multifaceted analyses in various biological studies.

## Materials and methods

### Genome assembly and annotation

Fresh *S. gemmata* leaves were harvested from Xinyang City, Henan Province (114.08°E, 32.13°N). Immediately after collection, they were rapidly frozen in liquid nitrogen and stored in a -80℃ environment, followed by DNA extraction. The libraries were constructed using the Nextera DNA Flex Library Prep Kit (Illumina, San Diego, CA, USA) and sequenced on the Illumina NovaSeq 6000 platform. The long fragment library was constructed using the SQK-LSK109 kit, quality-checked using Qubit, and then sequenced using the PromethION P48 platform.

Mitochondrial genome assembly and annotation:

1. The long-read data were assembled using the default parameters of Flye (v2.9) [55] to obtain graphical assembly results in GFA format. A library of all assembled contig sequences in GFA format was built using Makeblastdb, a specific module within BLAST (v2.13.0) [56], and contig fragments containing mitochondrial genomes were identified using the BLASTn (v2.13.0) [57], with conserved mitochondrial genes in Arabidopsis as query sequences with the parameters “-evalue 1e-5 -outfmt 6 -max_hsps 10 -word_size 7 -task blastn-short”. Visualization of GFA files was accomplished through Bandage (v0.8.1) [58]. Finally, the long-reads were mapped onto the graphical mitochondrial genome fragments using BWA (v0.7.17) [59] with default parameters, and the mitochondrial long-reads were exported for subsequent resolution of the repetitive sequence regions of the graphical plant mitochondrial genome.
2. Using the contigs obtained in step (1), we mapped short-reads to mitochondrial contigs using BWA (v0.7.17) with default parameters [59] and exported the mitochondrial short-reads. Based on the obtained mitochondrial short-reads and long-reads, we used Unicycler (Pacific Biosciences, Menlo Park, CA, USA) [60] for hybrid assembly to obtain the final mitochondrial genome sequence.
3. Mitochondrial genes of Stewartia gemmata were annotated utilizing Geseq v2.03 (https://chlorobox.mpimp-golm.mpg.de/geseq.html) [61]; We employed homologous comparison with mitochondrial genomes from four other reference species. The reference mitochondrial genomes included Arabidopsis thaliana (NC_037304), known for its complete and well-curated mitochondrial genes, Liriodendron tulipifera (NC_021152.1), recognized for its comprehensive mitochondrial variable genes, along with Camellia nitidissima (NC_067639.1) and Camellia sinensis (NC_043914.1), both closely related to Stewartia gemmata within the Theaceae family, to ensure precise and accurate annotation of mitochondrial genes. tRNAs were annotated using tRNAscan-SE (v2.0.11) [62], and rRNAs were annotated using BLASTn (v2.13.0) [57]. Each gene was manually corrected using Apollo (v1.11.8) [63].

Chloroplast genome assembly and annotation:

- The sequencing data were assembled using the default parameters of GetOrganelle (v1.7.5) [64] with the parameter “-R 15 -k 21,45,65,85,105 -F embplant_pt”, resulting in a cyclic S. gemmata chloroplast genome. The chloroplast genome was annotated using CPGAVAS2 (http://47.96.249.172:16019/analyzer/home, last accessed on April 2023) [65]. tRNA was annotated using tRNAscan-SE (v.2.0.11) [62], and rRNA was annotated using BLASTn (v2.13.0) [57]. Annotation errors for each gene were manually modified using CPGView (http://www.1kmpg.cn/pmgview) [66] and Apollo (v1.11.8) [63]. OGdraw (https://chlorobox.mpimp-golm.mpg.de/OGDraw.html) [67] was used to map the structure of the mitochondrial genome and the chloroplast genome, and the chloroplast and mitochondrial genome sequences are stored in GenBank, with the accession numbers OQ948158 (Mitochondrial genome) and OQ948159 (Chloroplast genome).

### Estimation of nucleotide substitution rates between homologous gene pairs

We used Phylosuite (v1.1.16) [68] to extract orthologous genes from collected mitochondrial and chloroplast genome datasets; and performed multiple sequence alignments by using MAFFT (v7.505, parameter “–auto”) [69]. The nucleotide substitution rates of paired orthologous genes, including the nonsynonymous mutation rate (*d*_N_), synonymous mutation rate (*d*_S_), and *d*_N_ to *d*_S_ ratio, were estimated using the yn00 module in pamlX (v1.3.1) [70]. The pairwise nonsynonymous (*d*_N_), synonymous (*d*_S_), and the ratio of nonsynonymous to synonymous substitutions (*d*_N_/*d*_S_) were visualized using density plots for each paired orthologs generated by the ggplot2 [71] package in R.

### Codon usage bias analysis

Protein-coding sequences of the genome were extracted using Phylosuite (v1.1.16) [68]. Protein-coding genes of the mitochondrial genome were analyzed for codon preference using MEGA (v7.0) [72], and RSCU values were calculated. Effective codon number (ENC) values and synonymous mutation codon position 3 GC content (GC3s) were calculated using CodonW (v1.4.4) [73]. All data were visualized the R-package ggplot2 [71].

### RNA editing site identification

We used the sequences of all protein-coding genes (PCGs) encoded by the mitochondrial genome of this species as input files and used Deepred-mt [74] to predict the C to U RNA editing sites of mitochondrial PCGs. We then retained all results with probability values greater than 0.9.

### Repeat sequence analysis

The sequences, including microsatellite sequence repeats and tandem repeats, were identified using MISA (v2.1) (https://webblast.ipk-gatersleben.de/misa/) [75], TRF (v4.09) (https://tandem.bu.edu/trf/trf.unix.help.html) [76], and the REPuter web server (https://bibiserv.cebitec.uni-bielefeld.de/reputer/) [77]. The results were visualized using Excel (2021) and Circos (v0.69.9) [78].

### Analysis of lateral gene transfer and TE identification

The corresponding positions of LGT sequences were obtained by comparing the chloroplast genome with the mitochondrial genome using BLASTn (v2.13.0) and default parameters [57], and the analysis of lateral gene transfer results was visualized using the Circos package (v0.69.9) [78]. The CENSOR (https://www.girinst.org/censor/index.php) [79] was used to identify TEs in *S. gemmata* organelle genomes, with default parameters and "green plants" as reference.

### Phylogenetic analysis

Orthologous genes were extracted using PhyloSuite (v1.1.16) [68], multiple sequence alignment analysis was performed using MAFFT (v7.505, parameter “–auto”), followed by the concatenation of sequences into a super matix by using PhyloSuite, and finally ML phylogenetic analysis by using IQ-TREE(v1.6.12) with parameter “-m GTR + G -bb 1000”, and the phylogenetic tree was visualized using ITOL (v6) [80]. All genomic data were obtained from the NCBI GenBank database. Details were recorded in Supplementary Table 15.

### Gene colinearity analysis

We selected the mitochondrial genomes of representative Theaceae species with accession IDs OL989850.1 [30], MH376284.1 [81], MK574876.1 and MK574877.1 [82], OP270590.1 [83], NC067639.1, and OM809792.1, and all genomic data were obtained from the NCBI GenBank database. BLASTn results for a two-by-two comparison of individual mitochondrial genomes were obtained based on the BLAST (v2.13.0) [57] program, and homologous sequences longer than 500 bp were retained as conserved colinear blocks. A multiple synteny plot was plotted using MCScanX [84].

### Supplementary Information

**Additional file 1:** **Supplementary Figure 1.** Construction of the mitochondrial genome assembly graph for *Stewartia gemmata*. (A) illustrates the preliminary draft of the mitochondrial genome. (B) describes the major circular conformation of the mitochondrial genome, while (C) explores potential alternative conformations of (B). **Supplementary Figure 2.** Depth of coverage of organelle genomes by sequenced sequences. (A), (B), and (C) show the depth of coverage of the genome by short-reads and long-reads mapped to the mitochondrial genome and short-reads mapped to the chloroplast genome, respectively. **Supplementary Figure 3.** Comparative analysis of the gene content in mitochondrial genomes of representative species in Ericales. Displayed are protein-coding genes (A), rRNA genes (B), and tRNA genes (C). Yellow means one copy exists; white means no copy exists. The red font indicates the genome released in this study. **Supplementary Figure 4.** Comparative analysis of the gene content in chloroplast genomes of representative species in Ericales. Displayed are protein-coding genes (A), trRNA genes (B), and rRNA genes (C). Yellow means a copy exists, white means no copy exists. The red font indicates the genome released in this study. **Supplementary Figure 5.** Distribution of long-reads mapping of the MTPT (Mitochondrial plastid DNAs) region of the mitochondrial genome. The long-reads from chloroplasts and mitochondria are mapped onto the mitochondrial genome, and the corresponding regions mapped are observed. In the figure, mitochondrial reads can map to the MTPT and regions on both sides, while chloroplast reads can only map to the MTPT region and not to the regions on both sides. MTPTs below 80 bp in length are not shown here due to their short length. For each MTPT, the gray area indicates the region covered by the sequence and the dark blue line segments highlight the mitochondrial (top) and chloroplast (bottom) reads, respectively, as representative of the two reads. **Supplementary Figure 6.** Best maximum likelihood phylogenetic tree based on mitochondrial genomes of 36 species (including seven species of Theaceae). Numbers above each branch are the maximum likelihood bootstrap of each clade >50%. The four different background colors indicate different orders, respectively. Red font indicates the species of this study. The top right corner is a tree with branch length information.

** Additional file 2:** **Supplementary Table 1.** Long-read coverage of possible conformational pathways in the *S. gemmata* genome. **Supplementary Table 2.** Summary of genomic information of *S. gemmata* organelles. **Supplementary Table 3.** Gene contents of the mitochondrial genome of *S. gemmata*. **Supplementary Table 4.** Gene contents of the chloroplast genome of *S. gemmata*. **Supplementary Table 5.** Relative synonymous codon usage (RSCU) statistics for individual amino acids in *S. gemmata* mitochondrial and chloroplast genomes. **Supplementary Table 6.** Statistics of ENC, GC3 values of individual genes in *S. gemmata* mitochondrial and chloroplast genomes. "-" indicates null values, red numbers are genes with ENC values less than 35. **Supplementary Table 7.** The information table for S. gemmata mitochondrial genomic RNA editing sites. ❆indicates the acquisition of the start codon, ✱indicates the acquisition of the stop codon. **Supplementary Table 8.** SSRs in the organelle genome of *S. gemmata*. p1, p2, p3, p4, p5, p6 in SSR type means Monomeric, Dimeric, Trimeric, Tetrameric, Pentameric, Hexamer. **Supplementary Table 9.** Tandem repeat sequences in the organelle genome of *S. gemmata*. **Supplementary Table 10.** Dispersed repeat sequences in the organelle genome of *S. gemmata*. **Supplementary Table 11.** Mitochondrial plastid DNAs (MTPTs) were identified in the mitochondrial genome of *S. gemmata*. **Supplementary Table 12.** Classification and characterization of Transposable Element (TE) fragments in the genome of *S. gemmata*. **Supplementary Table 13.** TE annotation in the organelle genome of *S. gemmata*. **Supplementary Table 14.** Overlap region between TEs and MTPTs in S. gemmata organelle genomes. The asterisk (*) symbols denote TEs found in the two types of organelle genomes. **Supplementary Table 15.** Species used to construct phylogenetic relationships. **Supplementary Table 16.** Colinear analysis of the mitochondrial genomes of seven species of Theaceae, including *Stewartia gemmata*.

## Data Availability

The complete organelle genomic sequence and annotations of *Stewartia gemmata* are available in the National Center for Biotechnology Information (NCBI) GenBank database (https://www.ncbi.nlm.nih.gov/genbank/), and the mitochondrial and chloroplast genomes can be accessed under the accession numbers OQ948158 and OQ948159, respectively. All sequencing data are stored in GenBank, BioProject accession No. PRJNA991307, including the sequencing sequence of two types of organelle genomes (SRR25132276 to SRR25132278).

## References

[1] Cheng L, Li M, Han Q, Qiao Z, Hao Y, Balbuena TS, Zhao Y (2022). Phylogenomics resolves the phylogeny of Theaceae by using low-copy and multi-copy nuclear gene makers and uncovers a fast radiation event contributing to tea plants diversity. Biology-Basel.

[2] Ming TL, Bartholomew B: *Stewartia*. In: *Flora of China vol.*, vol. pp: Science Press, Beijing, and Missouri Botanical Garden Press, St Louis; 2007: 424–429.

[3] Luna I, Ochoterena H (2004). Phylogenetic relationships of the genera of Theaceae based on morphology. Cladistics.

[4] Li J, Del Tredici P, Yang S, Donoghue MJ (2002). Phylogenetic relationships and biogeography of *Stewartia* (Camellioideae, Theaceae) inferred from nuclear ribosomal DNA ITS sequences. Rhodora.

[5] Gong W, Xiao S, Wang L, Liao Z, Chang Y, Mo W, Hu G, Li W, Zhao G, Zhu H (2022). Chromosome-level genome of *Camellia lanceoleosa* provides a valuable resource for understanding genome evolution and self-incompatibility. PLANT J.

[6] Lin P, Wang K, Wang Y, Hu Z, Yan C, Huang H, Ma X, Cao Y, Long W, Liu W (2022). The genome of oil-Camellia and population genomics analysis provide insights into seed oil domestication. Genome Biol.

[7] Shen TF, Huang B, Xu M, Zhou PY, Ni ZX, Gong C, Wen Q, Cao FL, Xu LA (2022). The reference genome of Camellia chekiangoleosa provides insights into Camellia evolution and tea oil biosynthesis. Hortic Res.

[8] Wei C, Yang H, Wang S, Zhao J, Liu C, Gao L, Xia E, Lu Y, Tai Y (2018). Draft genome sequence of *Camellia sinensis* var. *sinensis* provides insights into the evolution of the tea genome and tea quality. Proc Natl Acad Sci U S A.

[9] Zhang X, Chen S, Shi L, Gong D, Zhang S, Zhao Q, Zhan D, Vasseur L, Wang Y, Yu J (2021). Haplotype-resolved genome assembly provides insights into evolutionary history of the tea plant *Camellia sinensis*. Nat Genet.

[10] Chen JD, Zheng C, Ma JQ, Jiang CK, Ercisli S, Yao MZ, Chen L (2020). The chromosome-scale genome reveals the evolution and diversification after the recent tetraploidization event in tea plant. Hortic Res-England.

[11] Møller IM, Rasmusson AG, Van Aken O (2021). Plant mitochondria - past, present and future. Plant J.

[12] Maréchal A, Brisson N (2010). Recombination and the maintenance of plant organelle genome stability. New Phytol.

[13] Wolfe KH, Li WH, Sharp PM (1987). Rates of nucleotide substitution vary greatly among plant mitochondrial, chloroplast, and nuclear DNAs. P Natl Acad Sci USA.

[14] Moore LJ, Coleman AW (1989). The linear 20 kb mitochondrial genome of *Pandorina morum* (Volvocaceae, Chlorophyta). Plant Mol Biol.

[15] Sloan DB, Alverson AJ, Chuckalovcak JP, Wu M, McCauley DE, Palmer JD, Taylor DR (2012). Rapid evolution of enormous, multichromosomal genomes in flowering plant mitochondria with exceptionally high mutation rates. PLOS Biol.

[16] Palmer JD, Herbon LA (1988). Plant mitochondrial DNA evolves rapidly in structure, but slowly in sequence. J Mol Evol.

[17] Alverson AJ, Zhuo S, Rice DW, Sloan DB, Palmer JD (2011). The mitochondrial genome of the legume *Vigna radiata* and the analysis of recombination across short mitochondrial repeats. PLoS One.

[18] Xia C, Li J, Zuo Y, He P, Zhang H, Zhang X, Wang B, Zhang J, Yu J, Deng H (2023). Complete mitochondrial genome of *Thuja sutchuenensis* and its implications on evolutionary analysis of complex mitogenome architecture in Cupressaceae. BMC Plant Biol.

[19] Sloan DB (2013). One ring to rule them all? Genome sequencing provides new insights into the 'master circle' model of plant mitochondrial DNA structure. New Phytol.

[20] Zupok A, Kozul D, Schöttler MA, Niehörster J, Garbsch F, Liere K, Fischer A, Zoschke R, Malinova I, Bock R (2021). A photosynthesis operon in the chloroplast genome drives speciation in evening primroses.

[21] Schmidt SB, Eisenhut M, Schneider A (2020). Chloroplast transition metal regulation for efficient photosynthesis. Trends Plant Sci.

[22] Daniell H, Lin CS, Yu M, Chang WJ (2016). Chloroplast genomes: diversity, evolution, and applications in genetic engineering. Genome Biol.

[23] Li J, Li J, Ma Y, Kou L, Wei J, Wang W (2022). The complete mitochondrial genome of okra (*Abelmoschus esculentus*): using nanopore long reads to investigate gene transfer from chloroplast genomes and rearrangements of mitochondrial DNA molecules. BMC Genom.

[24] Shearman JR, Sonthirod C, Naktang C, Pootakham W, Yoocha T, Sangsrakru D, Jomchai N, Tragoonrung S, Tangphatsornruang S (2016). The two chromosomes of the mitochondrial genome of a sugarcane cultivar: assembly and recombination analysis using long PacBio reads. Sci Rep-UK.

[25] Ruhfel BR, Gitzendanner MA, Soltis PS, Soltis DE, Burleigh JG (2014). From algae to angiosperms-inferring the phylogeny of green plants (*Viridiplantae*) from 360 plastid genomes. BMC Evol Biol.

[26] Birky CW (1995). Uniparental inheritance of mitochondrial and chloroplast genes: mechanisms and evolution. P Natl Acad Sci USA.

[27] Moore MJ, Bell CD, Soltis PS, Soltis DE (2007). Using plastid genome-scale data to resolve enigmatic relationships among basal angiosperms. P Natl Acad Sci USA.

[28] Palmer JD: Mitochondrial DNA in plant systematics: applications and limitations. *Molecular Systematics of Plants* 1992:36–49.

[29] Davis CC, Xi Z, Mathews S (2014). Plastid phylogenomics and green plant phylogeny: almost full circle but not quite there. Bmc Biol.

[30] Li J, Tang H, Luo H, Tang J, Zhong N, Xiao L (2023). Complete mitochondrial genome assembly and comparison of *Camellia sinensis* var. *Assamica* cv. Duntsa. Front Plant Sci.

[31] Li Y, Li Z, Schuiteman A, Chase MW, Li J, Huang W, Hidayat A, Wu S, Jin X (2019). Phylogenomics of orchidaceae based on plastid and mitochondrial genomes. Mol Phylogenet Evol.

[32] Ni Y, Li J, Chen H, Yue J, Chen P, Liu C (2022). Comparative analysis of the chloroplast and mitochondrial genomes of *Saposhnikovia divaricata* revealed the possible transfer of plastome repeat regions into the mitogenome. BMC Genom.

[33] Li C, Zhou L, Nie J, Wu S, Li W, Liu Y, Liu Y (2023). Codon usage bias and genetic diversity in chloroplast genomes of *Elaeagnus* species (Myrtiflorae: Elaeagnaceae). Physiol Mol Biol Pla.

[34] Wright F (1990). The 'effective number of codons' used in a gene. Gene.

[35] Edera AA, Gandini CL, Sanchez-Puerta MV (2018). Towards a comprehensive picture of C-to-U RNA editing sites in angiosperm mitochondria. Plant Mol Biol.

[36] Unseld M, Marienfeld JR, Brandt P, Brennicke A (1997). The mitochondrial genome of *Arabidopsis thaliana* contains 57 genes in 366,924 nucleotides. Nat Genet.

[37] Handa H (2003). The complete nucleotide sequence and RNA editing content of the mitochondrial genome of rapeseed (*Brassica napus* L.): comparative analysis of the mitochondrial genomes of rapeseed and *Arabidopsis thaliana*. Nucleic Acids Res.

[38] Maumus F, Quesneville H (2014). Ancestral repeats have shaped epigenome and genome composition for millions of years in *Arabidopsis thaliana*. Nat Commun.

[39] Milligan BG, Hampton JN, Palmer JD (1989). Dispersed repeats and structural reorganization in subclover chloroplast DNA. Mol Biol Evol.

[40] Rodriguez-Moreno L, Gonzalez VM, Benjak A, Marti MC, Puigdomenech P, Aranda MA, Garcia-Mas J (2011). Determination of the melon chloroplast and mitochondrial genome sequences reveals that the largest reported mitochondrial genome in plants contains a significant amount of DNA having a nuclear origin. BMC Genom.

[41] Yang JB, Yang SX, Li HT, Yang J, Li DZ (2013). Comparative chloroplast genomes of *Camellia* species. PLoS ONE.

[42] Schnable PS, Ware D, Fulton RS, Stein JC, Wei F, Pasternak S, Liang C, Zhang J, Fulton L, Graves TA (2009). The B73 maize genome: complexity, diversity, and dynamics. Science.

[43] Du J, Grant D, Tian Z, Nelson RT, Zhu L, Shoemaker RC, Ma J (2010). SoyTEdb: a comprehensive database of transposable elements in the soybean genome. BMC Genom.

[44] Singh AK, Chaurasia S, Kumar S, Singh R, Kumari J, Yadav MC, Singh N, Gaba S, Jacob SR (2018). Identification, analysis and development of salt responsive candidate gene based SSR markers in wheat. BMC Plant Biol.

[45] Zhang W, Zhou J, Li Z, Wang L, Gu X (2007). Comparative analysis of codon usage patterns among mitochondrion, chloroplast and nuclear genes in *Triticum aestivum* L. J Integr Plant Biol.

[46] Chen SL, Lee W, Hottes AK, Shapiro L, McAdams HH (2004). Codon usage between genomes is constrained by genome-wide mutational processes. P Natl Acad Sci USA.

[47] Zhang Y, Shen Z, Meng X, Zhang L, Liu Z, Liu M, Zhang F, Zhao J (2022). Codon usage patterns across seven Rosales species. BMC Plant Biol.

[48] Liu Q, Xue Q (2005). Comparative studies on codon usage pattern of chloroplasts and their host nuclear genes in four plant species. J Genet.

[49] Sablok G, Nayak KC, Vazquez F, Tatarinova TV (2011). Synonymous codon usage, GC(3), and evolutionary patterns across plastomes of three pooid model species: emerging grass genome models for monocots. Mol Biotechnol.

[50] Chakraborty S, Yengkhom S, Uddin A (2020). Analysis of codon usage bias of chloroplast genes in Oryza species : Codon usage of chloroplast genes in *Oryza* species. Planta.

[51] Niu Y, Luo Y, Wang C, Liao W (2021). Deciphering codon csage patterns in genome of *Cucumis sativus* in comparison with nine species of Cucurbitaceae. Agronomy.

[52] Knoop V, Unseld M, Marienfeld J, Brandt P, Sünkel S, Ullrich H, Brennicke A (1996). copia-, gypsy- and LINE-Like retrotransposon fragments in the mitochondrial genome of *Arabidopsis thaliana*. Genetics.

[53] Yu X, Jiang W, Tan W, Zhang X, Tian X (2020). Deciphering the organelle genomes and transcriptomes of a common ornamental plant *Ligustrum quihoui* reveals multiple fragments of transposable elements in the mitogenome. Int J BioL Macromol.

[54] Yu XQ, Gao LM, Soltis DE, Soltis PS, Yang JB, Fang L, Yang SX, Li DZ (2017). Insights into the historical assembly of East Asian subtropical evergreen broadleaved forests revealed by the temporal history of the tea family. New Phytol.

[55] Kolmogorov M, Yuan J, Lin Y, Pevzner PA (2019). Assembly of long, error-prone reads using repeat graphs. Nat Biotechnol.

[56] Altschul S (1997). Gapped BLAST and PSI-BLAST: a new generation of protein database search programs. Nucleic Acids Res.

[57] Chen Y, Ye W, Zhang Y, Xu Y (2015). High speed BLASTN: an accelerated mega BLAST search tool. Nucleic Acids Res.

[58] Wick RR, Schultz MB, Zobel J, Holt KE (2015). Bandage: interactive visualization of de novo genome assemblies. Bioinformatics.

[59] Li H, Durbin R (2009). Fast and accurate short read alignment with Burrows-Wheeler transform. Bioinformatics.

[60] Wick RR, Judd LM, Gorrie CL, Holt KE (2017). Unicycler: resolving bacterial genome assemblies from short and long sequencing reads. PLOS Comput Biol.

[61] Tillich M, Lehwark P, Pellizzer T, Ulbricht-Jones ES, Fischer A, Bock R, Greiner S (2017). GeSeq - versatile and accurate annotation of organelle genomes. Nucleic Acids Res.

[62] Lowe TM, Eddy SR (1997). tRNAscan-SE: A program for improved detection of transfer RNA genes in genomic sequence. Nucleic Acids Res.

[63] Lewis S, Searle S, Harris N, Gibson M, Iyer V, Richter J, Wiel C, Bayraktaroglu L, Birney E, Crosby M (2002). Apollo: a sequence annotation editor. Genome Biol.

[64] Jin JJ, Yu WB, Yang JB, Song Y, DePamphilis CW, Yi TS, Li DZ (2020). GetOrganelle: a fast and versatile toolkit for accurate de novo assembly of organelle genomes. Genome Biol.

[65] Shi L, Chen H, Jiang M, Wang L, Wu X, Huang L, Liu C (2019). CPGAVAS2, an integrated plastome sequence annotator and analyzer. Nucleic Acids Res.

[66] Liu S, Ni Y, Li J, Zhang X, Yang H, Chen H, Liu C (2023). CPGView: A package for visualizing detailed chloroplast genome structures. Mol Ecol Resour.

[67] Greiner S, Lehwark P, Bock R (2019). OrganellarGenomeDRAW (OGDRAW) version 1.3.1: expanded toolkit for the graphical visualization of organellar genomes.. Nucleic Acids Res.

[68] Zhang D, Gao F, Jakovlić I, Zou H, Zhang J, Li WX, Wang GT (2020). PhyloSuite: An integrated and scalable desktop platform for streamlined molecular sequence data management and evolutionary phylogenetics studies. Mol Ecol Resour.

[69] Katoh K, Misawa K, Kuma KI, Miyata T (2002). MAFFT: a novel method for rapid multiple sequence alignment based on fast Fourier transform. Nucleic Acids Res.

[70] Yang Z (2007). PAML 4: phylogenetic analysis by maximum likelihood. Mol Biol Evol.

[71] Wickham H (2011). ggplot2. WIREs Comput Stat.

[72] Kumar S, Stecher G, Tamura K (2016). MEGA7: molecular evolutionary genetics analysis version 7.0 for bigger datasets. Mol Biol Evol.

[73] Grote A, Hiller K, Scheer M, Münch R, Nörtemann B, Hempel DC, Jahn D (2005). JCat: a novel tool to adapt codon usage of a target gene to its potential expression host. NUCLEIC ACIDS RES.

[74] Edera AA, Small I, Milone DH, Sanchez-Puerta MV (2021). Deepred-Mt: deep representation learning for predicting C-to-U RNA editing in plant mitochondria. Comput Biol Med.

[75] Beier S, Thiel T, Münch T, Scholz U, Mascher M (2017). MISA-web: a web server for microsatellite prediction. Bioinformatics.

[76] Benson G (1999). Tandem repeats finder: a program to analyze DNA sequences. Nucleic Acids Res.

[77] Kurtz S, Choudhuri JV, Ohlebusch E, Schleiermacher C, Stoye J, Giegerich R (2001). REPuter: the manifold applications of repeat analysis on a genomic scale. Nucleic Acids Res.

[78] Zhang H, Meltzer P, Davis S (2013). RCircos: an R package for Circos 2D track plots. BMC Bioinform.

[79] Kohany O, Gentles AJ, Hankus L, Jurka J (2006). Annotation, submission and screening of repetitive elements in Repbase: RepbaseSubmitter and Censor. BMC Bioinform.

[80] Nguyen L, Schmidt HA, von Haeseler A, Minh BQ (2015). IQ-TREE: a fast and effective stochastic algorithm for estimating maximum-likelihood phylogenies. Mol Biol Evol.

[81] Rawal HC, Kumar PM, Bera B, Singh NK, Mondal TK (2020). Decoding and analysis of organelle genomes of Indian tea (*Camellia assamica*) for phylogenetic confirmation. Genomics.

[82] Zhang F, Li W, Gao C, Zhang D, Gao L (2019). Deciphering tea tree chloroplast and mitochondrial genomes of *Camellia sinensis* var. *assamica*. Sci Data.

[83] Lu C, Gao L, Zhang Q: A high-quality genome assembly of the mitochondrial genome of the oil-tea Tree *Camellia gigantocarpa* (Theaceae). In: *Diversity.*, vol. 14; 2022.

[84] Wang Y, Tang H, DeBarry JD, Tan X, Li J, Wang X, Lee T, Jin H, Marler B, Guo H (2012). MCScanX: a toolkit for detection and evolutionary analysis of gene synteny and collinearity. Nucleic Acids Res.

