# Supplementary material for: Decoding the complete organelle genomic architecture of Stewartia gemmata: an early-diverging species in Theaceae
Source: BMC Genomics. 2024 Jan 25;25:114. doi: 10.1186/s12864-024-10016-8 (PMC10811901; doi:10.1186/s12864-024-10016-8)
Supplement: Supplementary file 1 — Additional file 1: Supplementary Figure 1. Construction of the mitochondrial genome assembly graph for Stewartia gemmata. (A) illustrates the preliminary draft of the mitochondrial genome. (B) describes the major circular conformation of the mitochondrial genome, while (C) explores potential alternative conformations of (B). Supplementary Figure 2. Depth of coverage of organelle genomes by sequenced sequences. (A), (B), and (C) show the depth of coverage of the genome by short-reads and long-reads mapped to the mitochondrial genome and short-reads mapped to the chloroplast genome, respectively. Supplementary Figure 3. Comparative analysis of the gene content in mitochondrial genomes of representative species in Ericales. Displayed are protein-coding genes (A), rRNA genes (B), and tRNA genes (C). Yellow means one copy exists; white means no copy exists. The red font indicates the genome released in this study. Supplementary Figure 4. Comparative analysis of the gene content in chloroplast genomes of representative species in Ericales. Displayed are protein-coding genes (A), trRNA genes (B), and rRNA genes (C). Yellow means a copy exists, white means no copy exists. The red font indicates the genome released in this study. Supplementary Figure 5. Distribution of long-reads mapping of the MTPT (Mitochondrial plastid DNAs) region of the mitochondrial genome. The long-reads from chloroplasts and mitochondria are mapped onto the mitochondrial genome, and the corresponding regions mapped are observed. In the figure, mitochondrial reads can map to the MTPT and regions on both sides, while chloroplast reads can only map to the MTPT region and not to the regions on both sides. MTPTs below 80 bp in length are not shown here due to their short length. For each MTPT, the gray area indicates the region covered by the sequence and the dark blue line segments highlight the mitochondrial (top) and chloroplast (bottom) reads, respectively, as representative of the two reads. Suppleme [file 12864_2024_10016_MOESM1_ESM.docx]

**
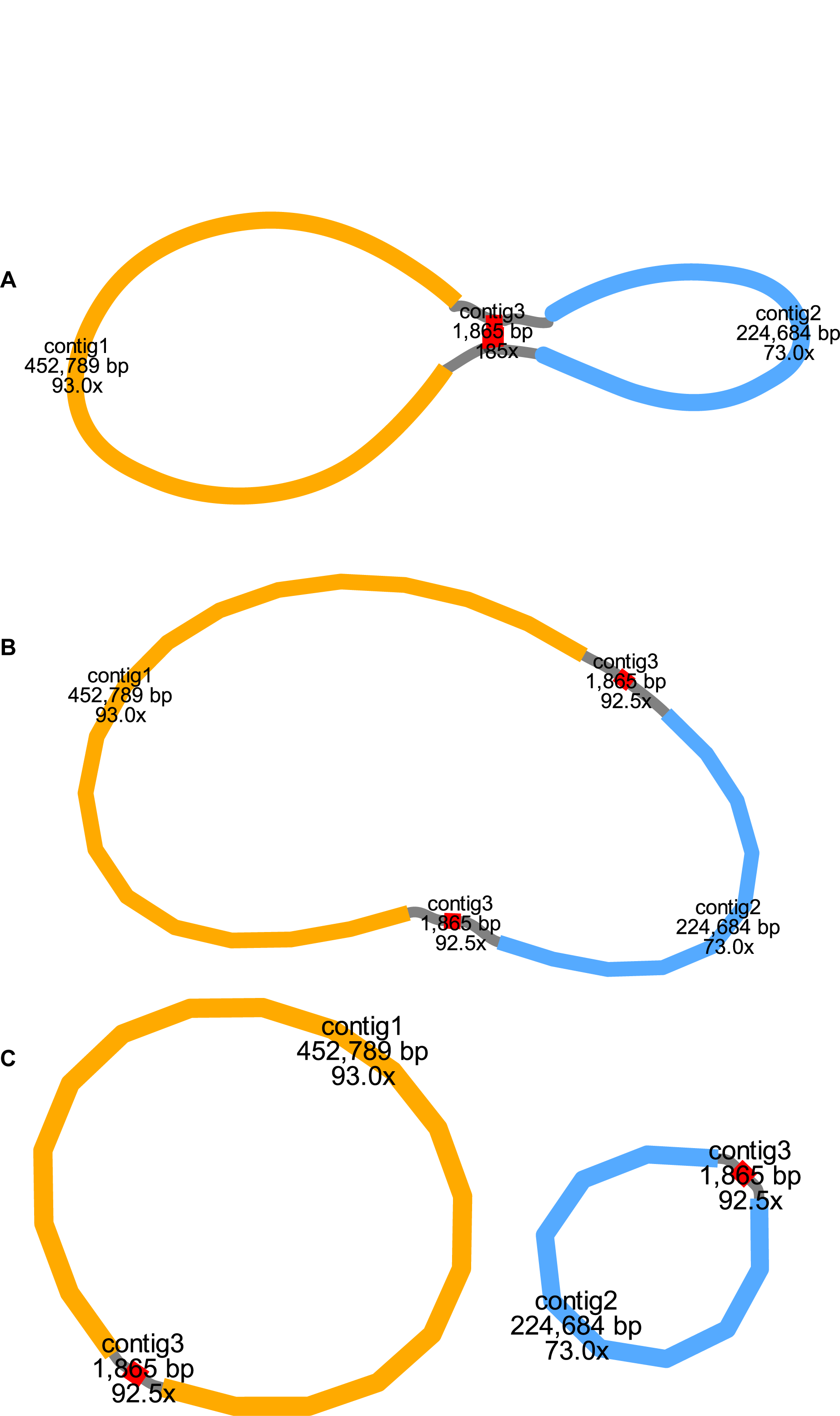
**

**Supplementary figure 1: Construction of the mitochondrial genome assembly graph for *Stewartia gemmata*.** (A) illustrates the preliminary draft of the mitochondrial genome. (B) describes the major circular conformation of the mitochondrial genome, while (C) explores potential alternative conformations of (B).

**
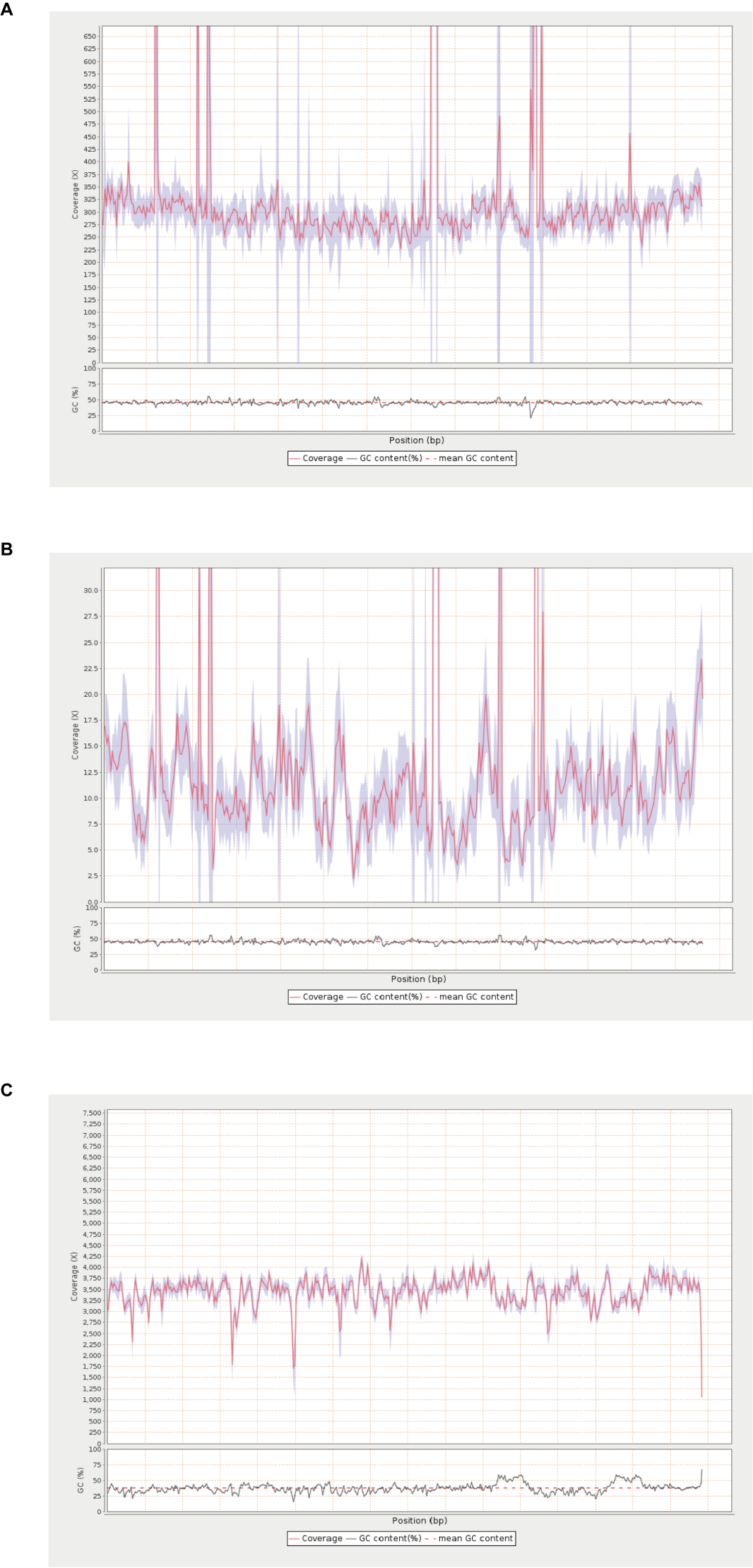
Supplementary figure 2: Depth of coverage of organelle genomes by sequenced sequences.** (A), (B), and (C) show the depth of coverage of the genome by short-reads and long-reads mapped to the mitochondrial genome and short-reads mapped to the chloroplast genome, respectively.

**
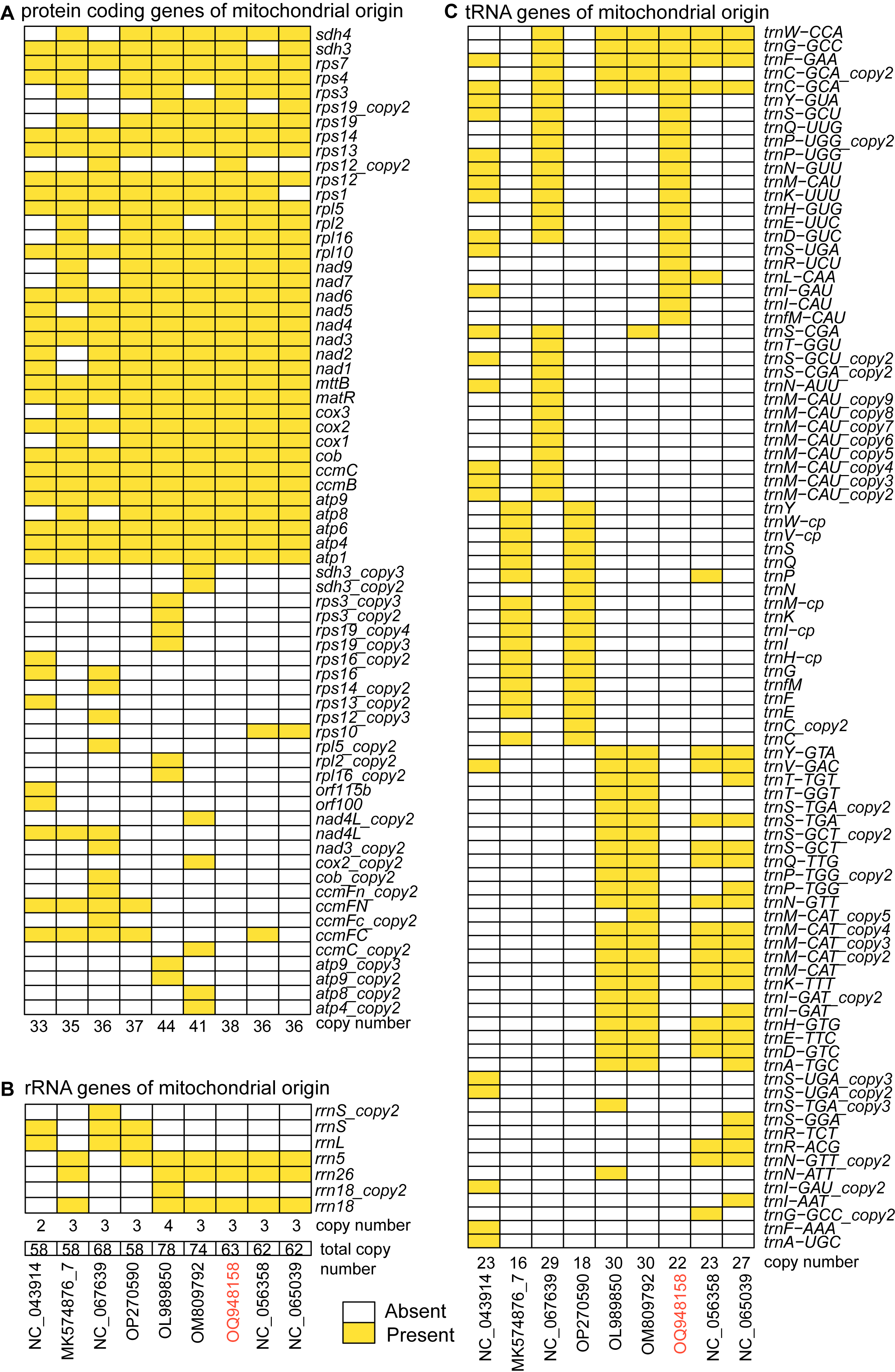
Supplementary figure 3: Comparative analysis of the gene content in mitochondrial genomes of representative species in Ericales.** Displayed are protein-coding genes (A), rRNA genes (B), and tRNA genes (C). Yellow means one copy exists; white means no copy exists. The red font indicates the genome released in this study.

**
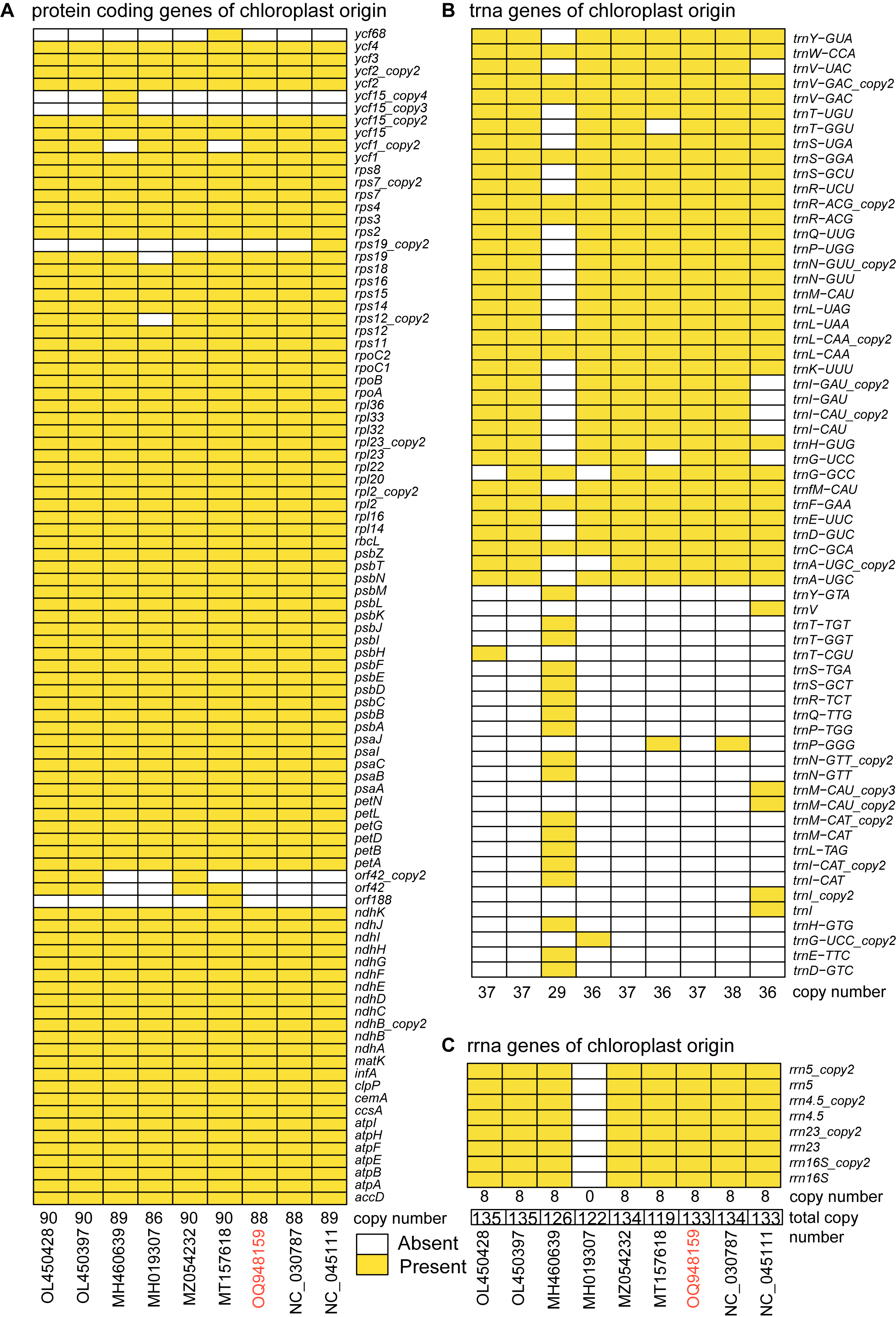
Supplementary figure 4: Comparative analysis of the gene content in chloroplast genomes of representative species in Ericales.** Displayed are protein-coding genes (A), trRNA genes (B), and rRNA genes (C). Yellow means a copy exists, white means no copy exists. The red font indicates the genome released in this study.

# MTPT1

mtDNA: 378,944-374,549

Length: 4,396 bp


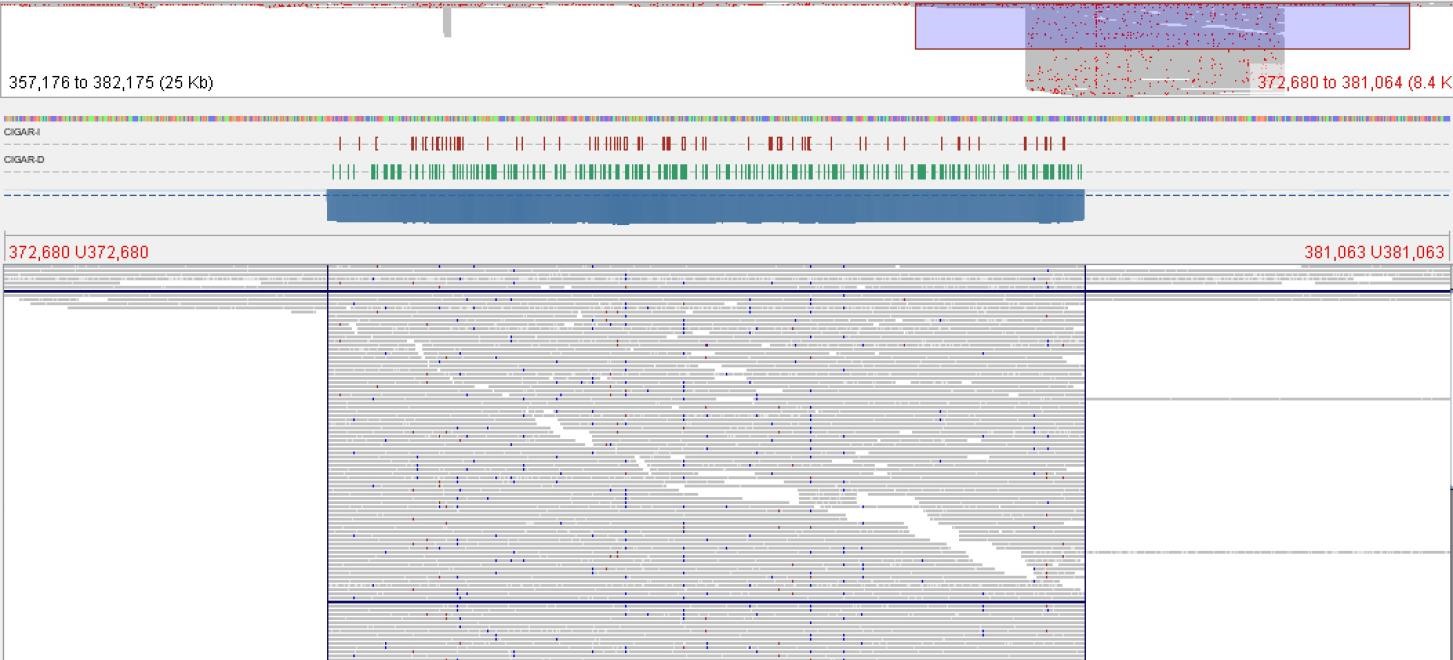


Plastid reads

Mitochondrial reads

MTPT1

# MTPT7

mtDNA: 351,440-351,357

Length: 84 bp


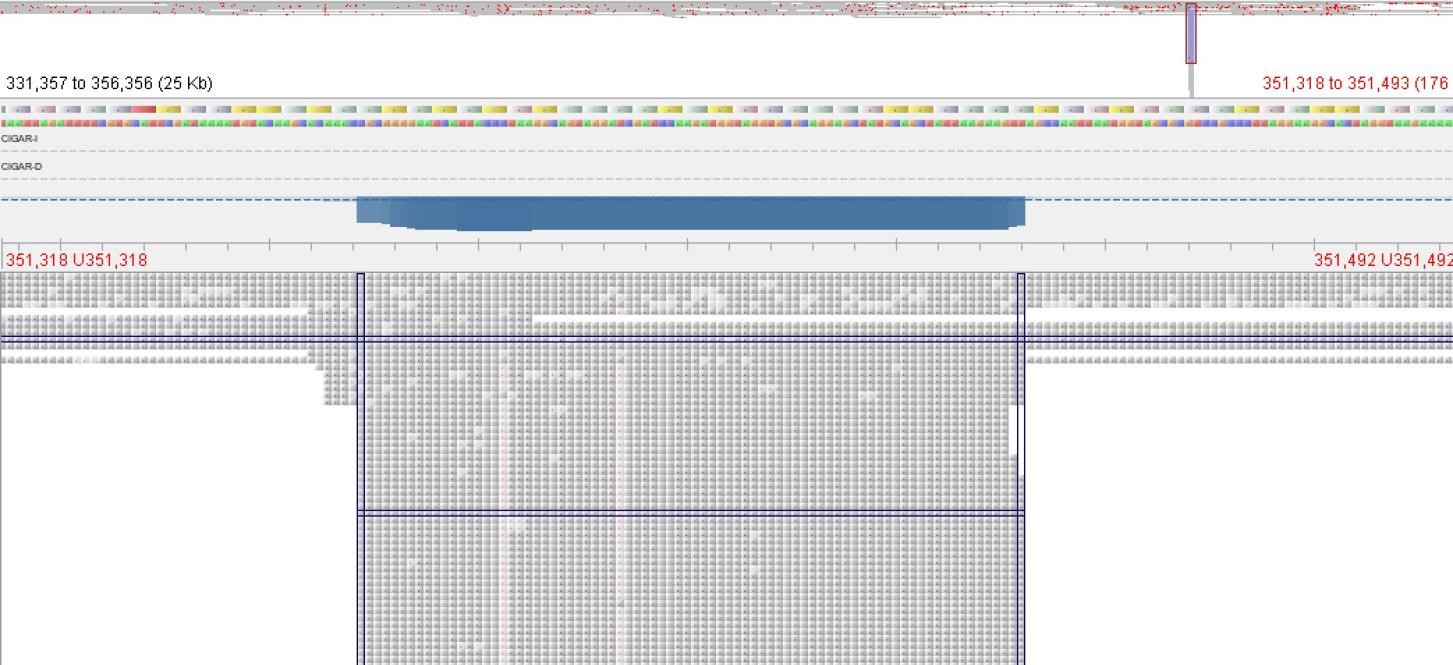


Mitochondrial reads

Plastid reads

MTPT7

# MTPT8

mtDNA: 364,660-364,809

Length: 150 bp


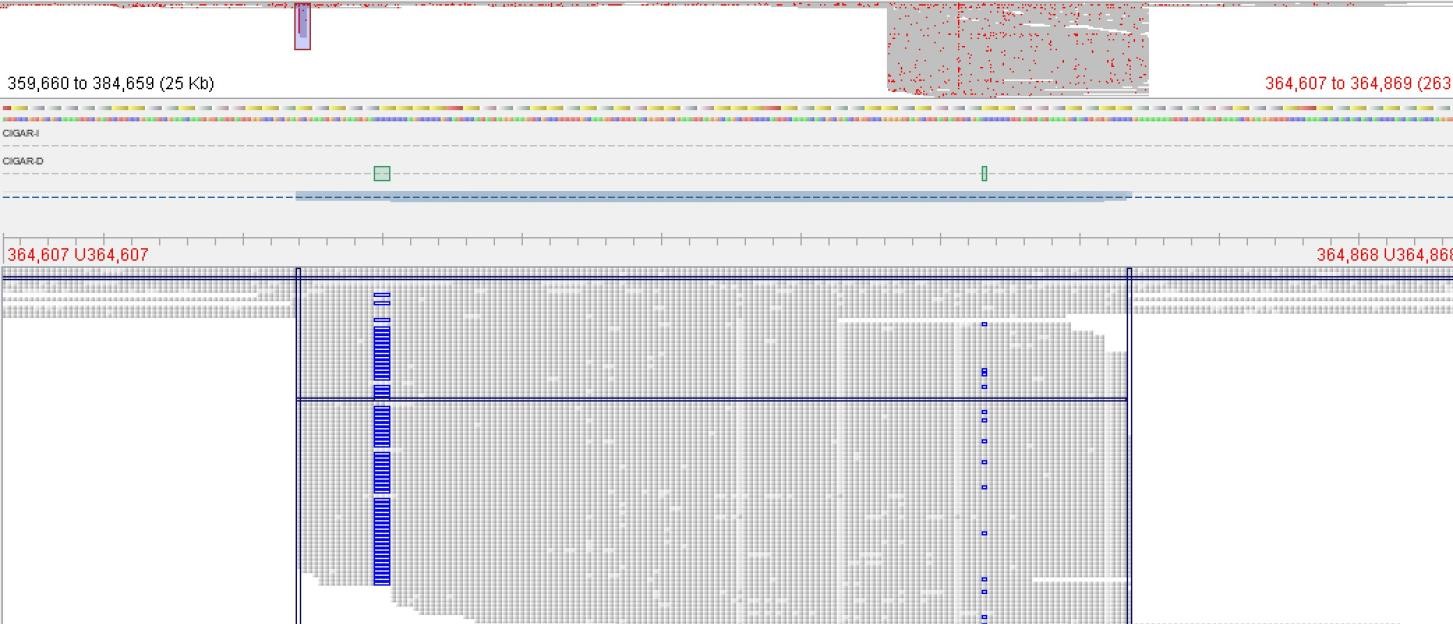


Mitochondrial reads

Plastid reads

MTPT8

# MTPT11

mtDNA: 198,580-198,699

Length: 126 bp


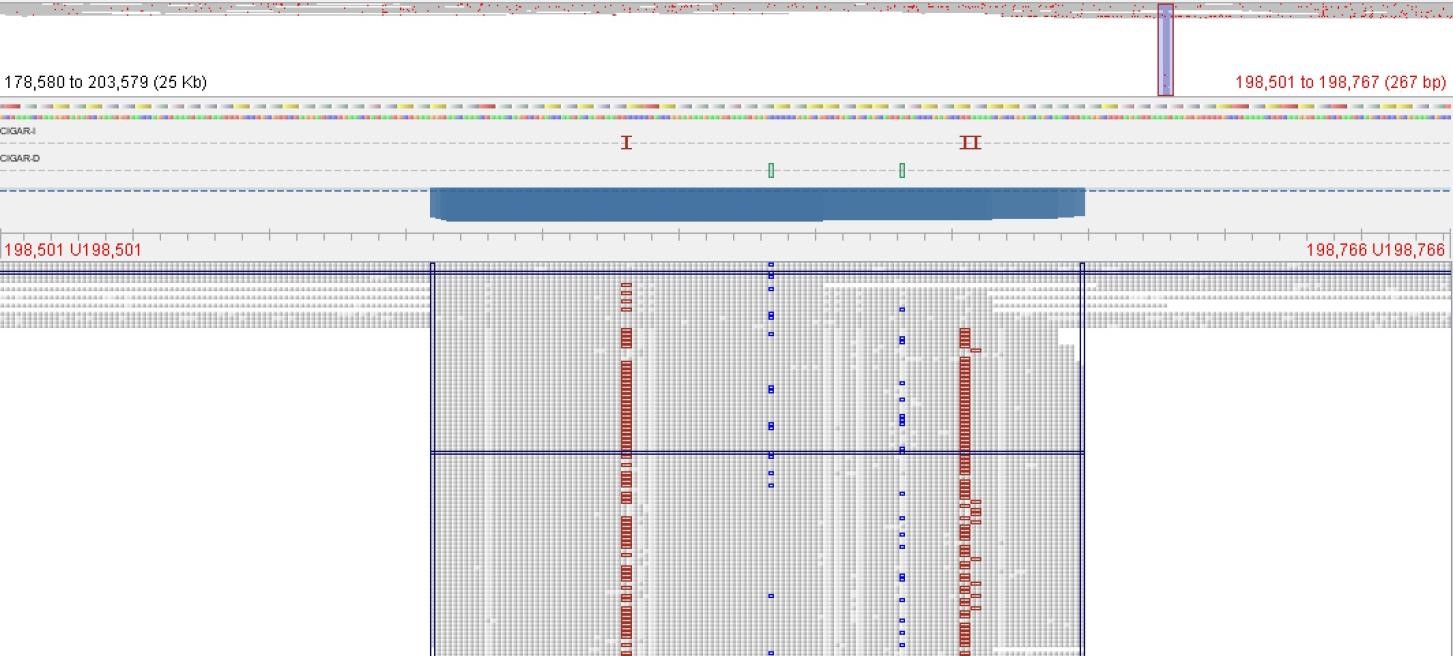


Mitochondrial reads

Plastid reads

MTPT11

# MTPT13

mtDNA: 487,235-487,425

Length: 196 bp


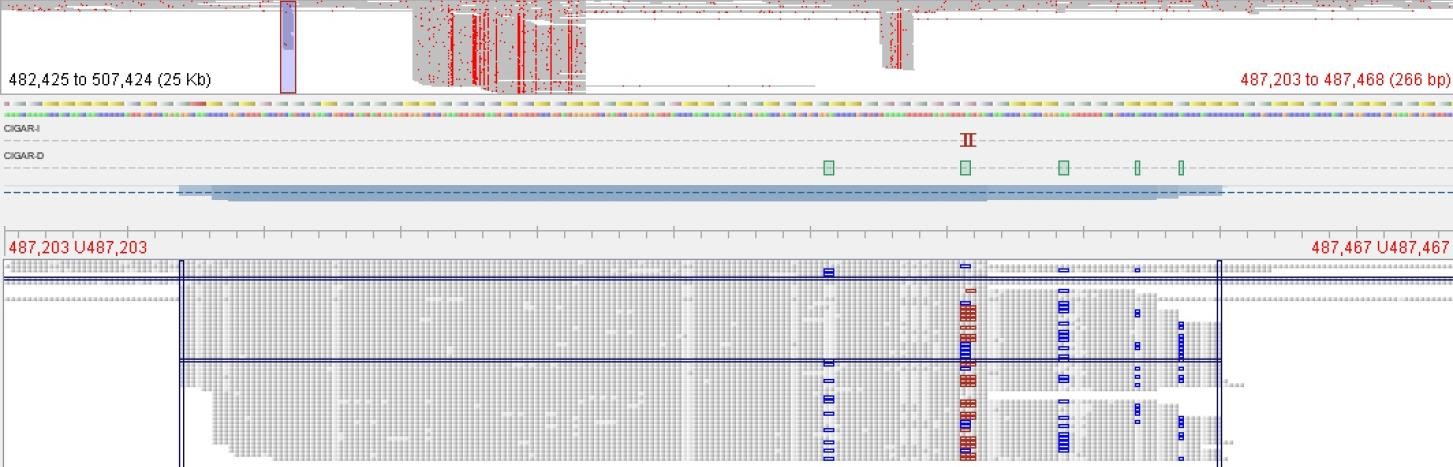


MTPT13

Mitochondrial reads

Plastid reads

# MTPT14

mtDNA: 108, 235-108,725

Length: 504 bp


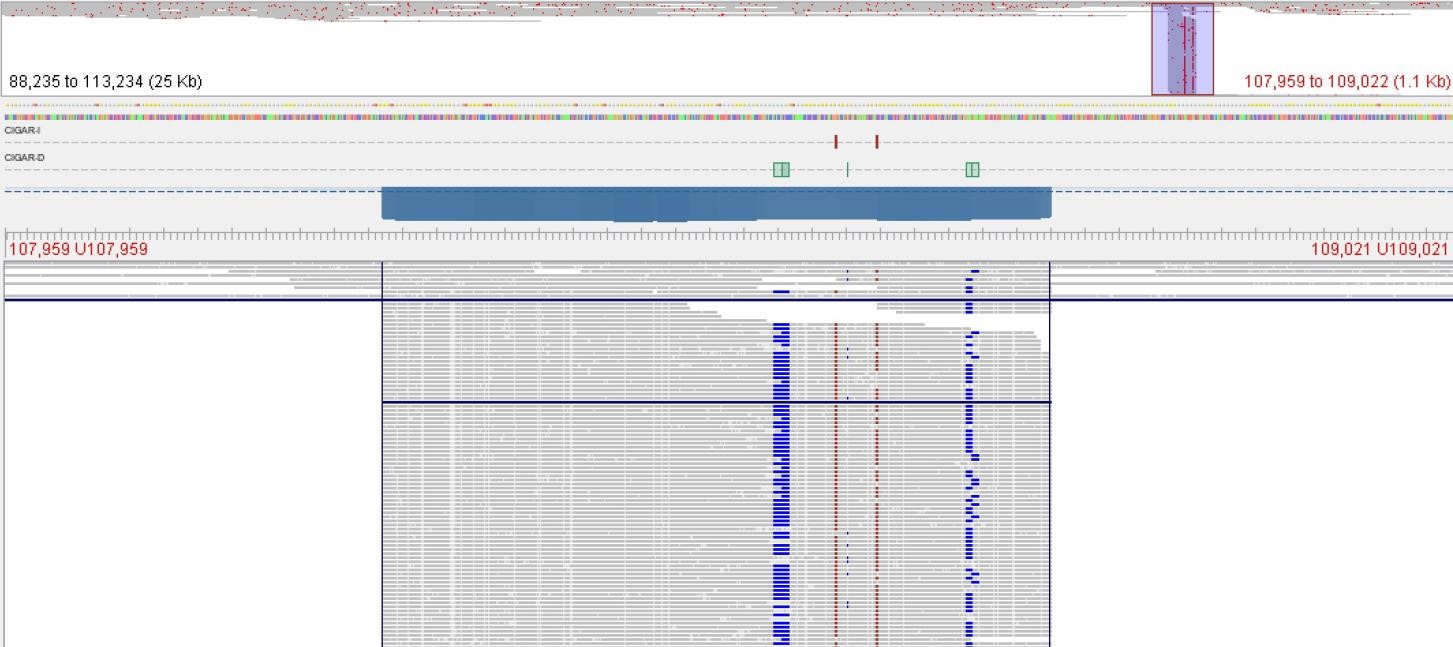


Mitochondrial reads

Plastid reads

MTPT14

# MTPT15

mtDNA: 60,056-61,513

Length: 1,506 bp


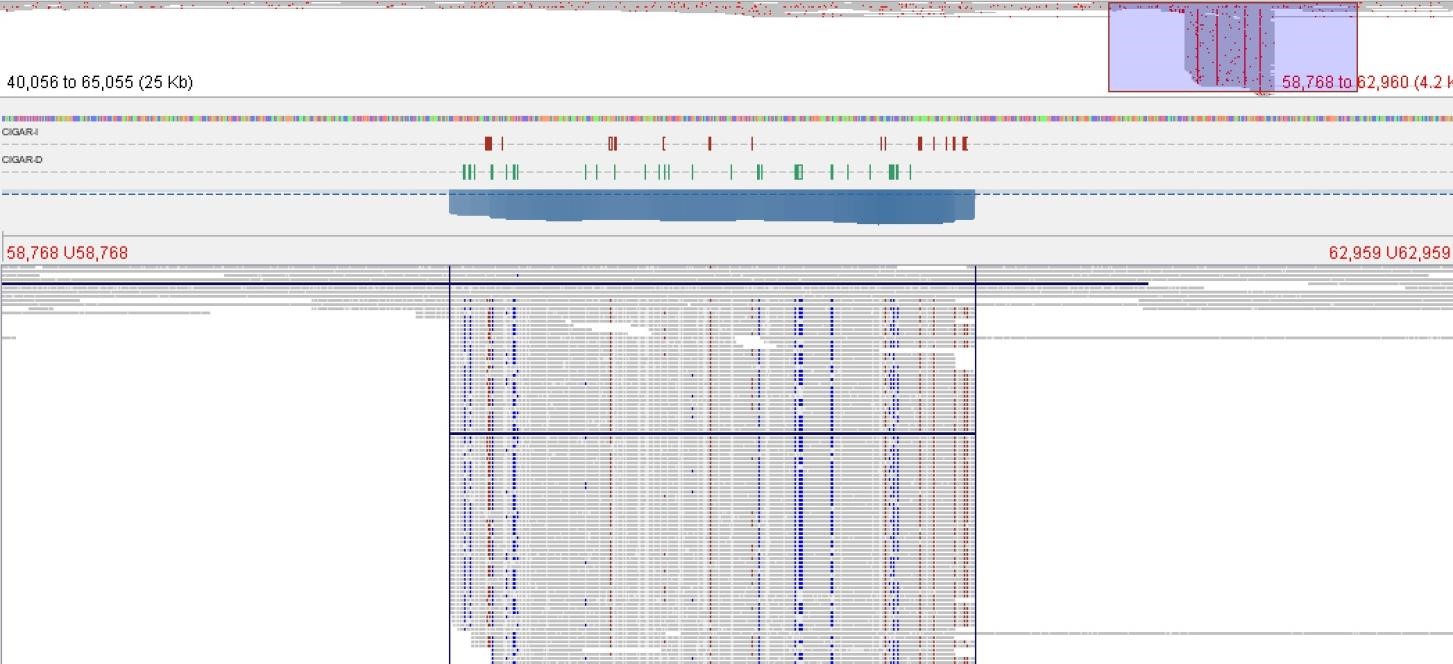


Mitochondrial reads

Plastid reads

MTPT15

# MTPT16

mtDNA: 497,650-498,057

Length: 414 bp


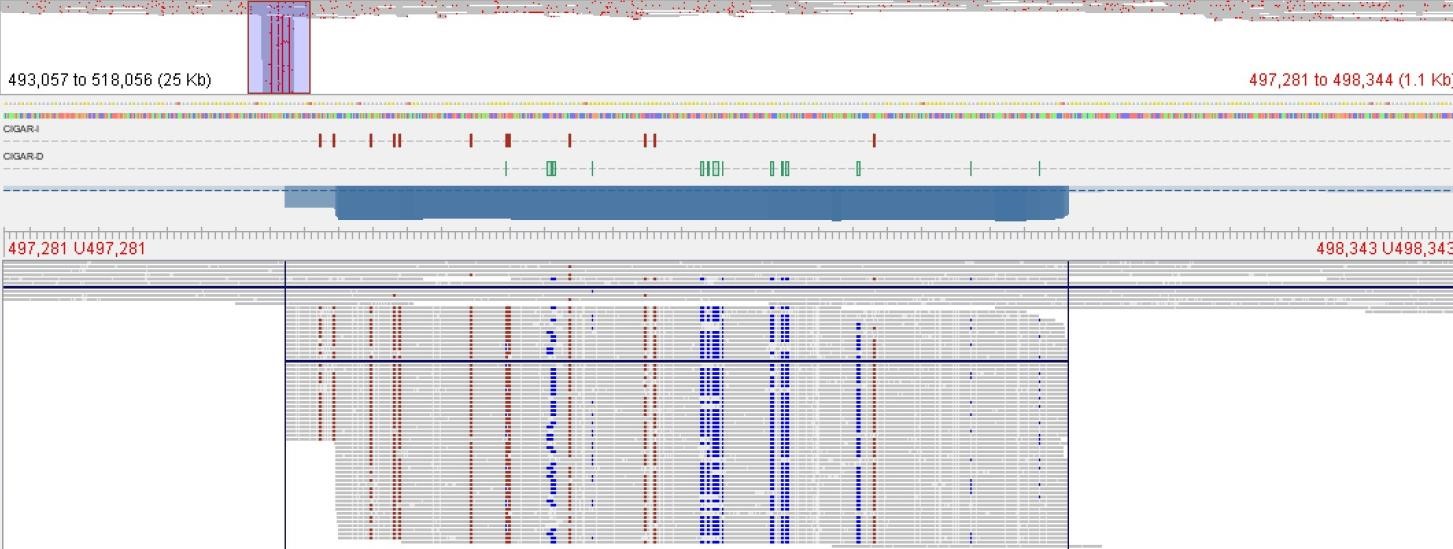


Mitochondrial reads

Plastid reads

MTPT16

# MTPT17

mtDNA: 492,451-489,462

Length: 3,123 bp


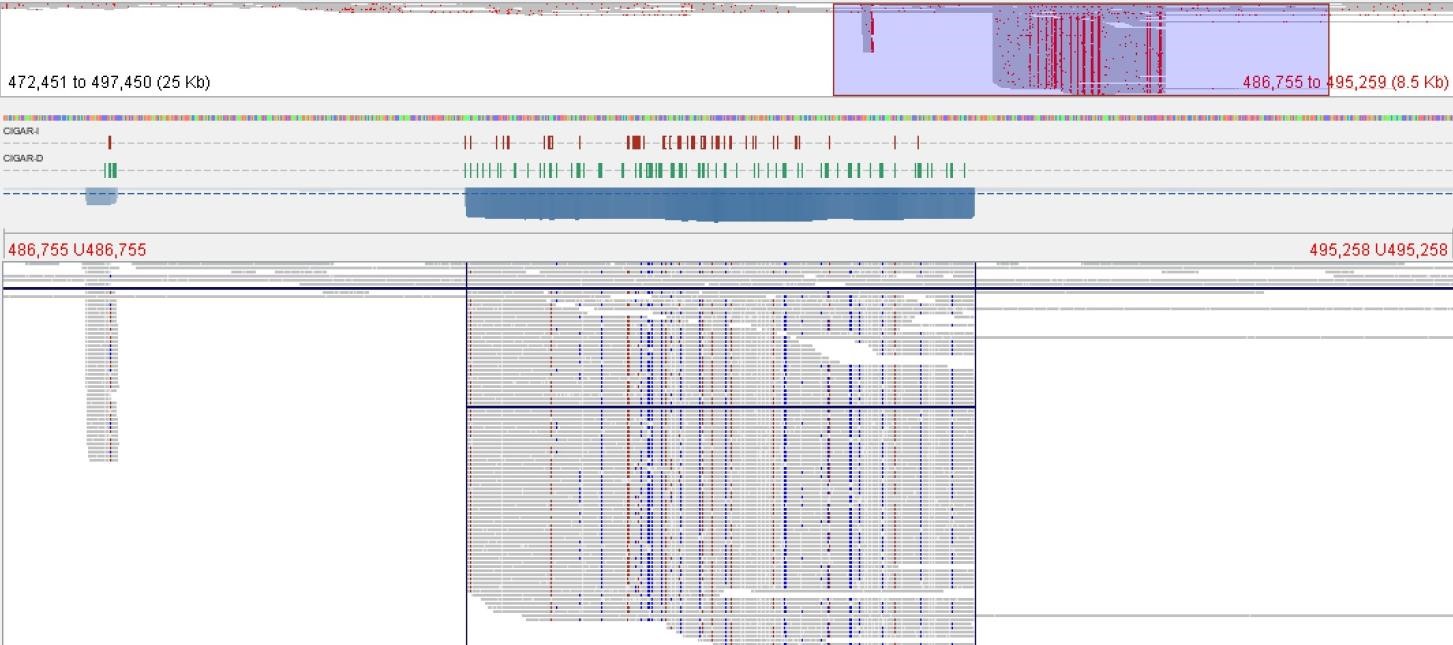


Mitochondrial reads

Plastid reads

MTPT17

**Supplementary figure 5: Distribution of long-reads mapping of the MTPT (Mitochondrial plastid DNAs) region of the mitochondrial genome.** The long-reads from chloroplasts and mitochondria are mapped onto the mitochondrial genome, and the corresponding regions mapped are observed. In the figure, mitochondrial reads can map to the MTPT and regions on both sides, while chloroplast reads can only map to the MTPT region and not to the regions on both sides. MTPTs below 80 bp in length are not shown here due to their short length. For each MTPT, the gray area indicates the region covered by the sequence and the dark blue line segments highlight the mitochondrial (top) and chloroplast (bottom) reads, respectively, as representative of the two reads.


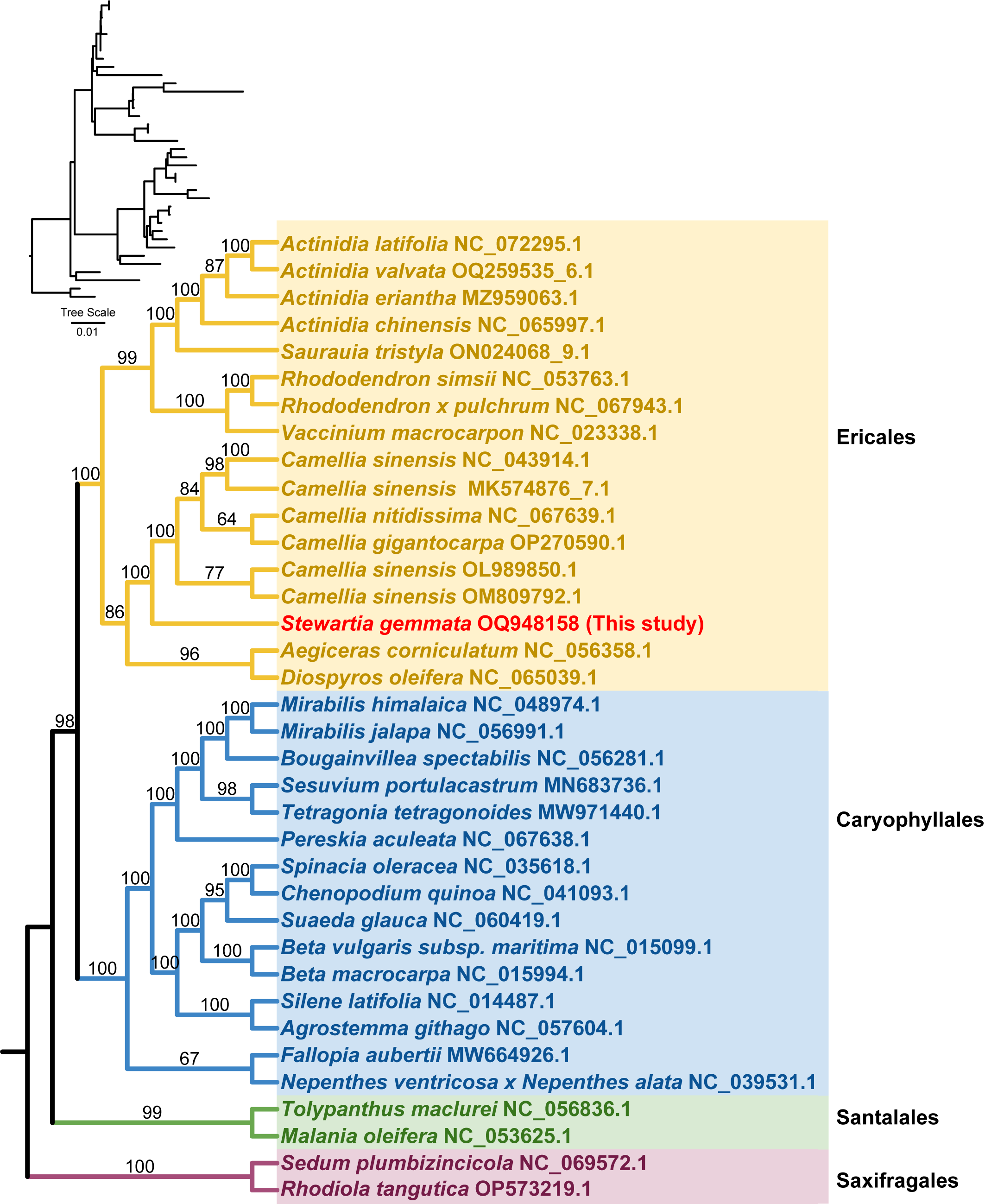


**Supplementary figure 6: Best maximum likelihood phylogenetic tree based on mitochondrial genomes of 36 species (including seven species of Theaceae).** Numbers above each branch are the maximum likelihood bootstrap of each clade >50%. The four different background colors indicate different orders, respectively. Red font indicates the species of this study. The top right corner is a tree with branch length information.
